# Comparative phytochemical profiling and authentication of four *Artemisia* species using integrated GC-MS, HPTLC and NIR spectroscopy approach

**DOI:** 10.1186/s13065-025-01467-5

**Published:** 2025-04-16

**Authors:** Ingy I. Abdallah, Hebaalla A. Mahmoud, Nadia A. El-Sebakhy, Yasmin A. Mahgoub

**Affiliations:** Department of Pharmacognosy, Faculty of Pharmacy, Alexandria University, Alexandria, 21521 Egypt

**Keywords:** *Artemisia annua*, *Artemisia herba-alba*, *Artemisia monosperma*, *Artemisia judaica*, Multivariate analysis, Phytochemical profile, Adulteration

## Abstract

Genus *Artemisia* has diverse phytochemistry and a long history in traditional medicine with several species still having unexplored potential. Hence, comparative profiling of *Artemisia* species in Egypt (*A. annua*, *A. herba-alba*, *A. monosperma* and *A. judaica*) and their authentication is of great interest. An integrated approach of GC-MS, HPTLC-image analysis and near-infrared (NIR) spectroscopy was implemented for their fingerprinting, discrimination and authentication. GC-MS analysis revealed the phytochemical profile of their volatile oils identifying compounds spanning monoterpenes, sesquiterpenes, diterpenes and non-terpenoid compounds. The major chemical components were highlighted as camphor, β-caryophyllene and germacrene D in *A. annua*, camphene, cis-pinocarveol, trans-chrysanthenyl acetate and cis-chrysanthenyl acetate in *A. herba-alba*, α-pinene, β-pinene, α-terpinolene and (-)-spathulenol in *A. monosperma*, finally, camphor, piperitone and trans-ethyl cinnamate in *A. judaica*. HPTLC-image analysis allowed tracking chemical markers in their total alcoholic extracts. Artemisinin was detected only in *A. annua* while scopoletin was identified as a major characteristic coumarin in *Artemisia* species. Phenolic acids and flavonoids were also discovered in the different species. Finally, NIR spectroscopy allowed profiling and authentication of their powders revealing prominent spectral characteristics correlated to the chemical markers identified by GC-MS and HPTLC. Then, multivariate analysis facilitated classification and discrimination of the species. Additionally, PLS regression analysis was utilized for quality control of powdered *A. annua*, being an important industrial crop, by detecting its adulteration with other species in limits of detection less than 1.5%. This combined approach aided in the rapid comparative profiling of the *Artemisia* species as a mean for their fingerprinting and authentication.

**Graphical Abstract:**

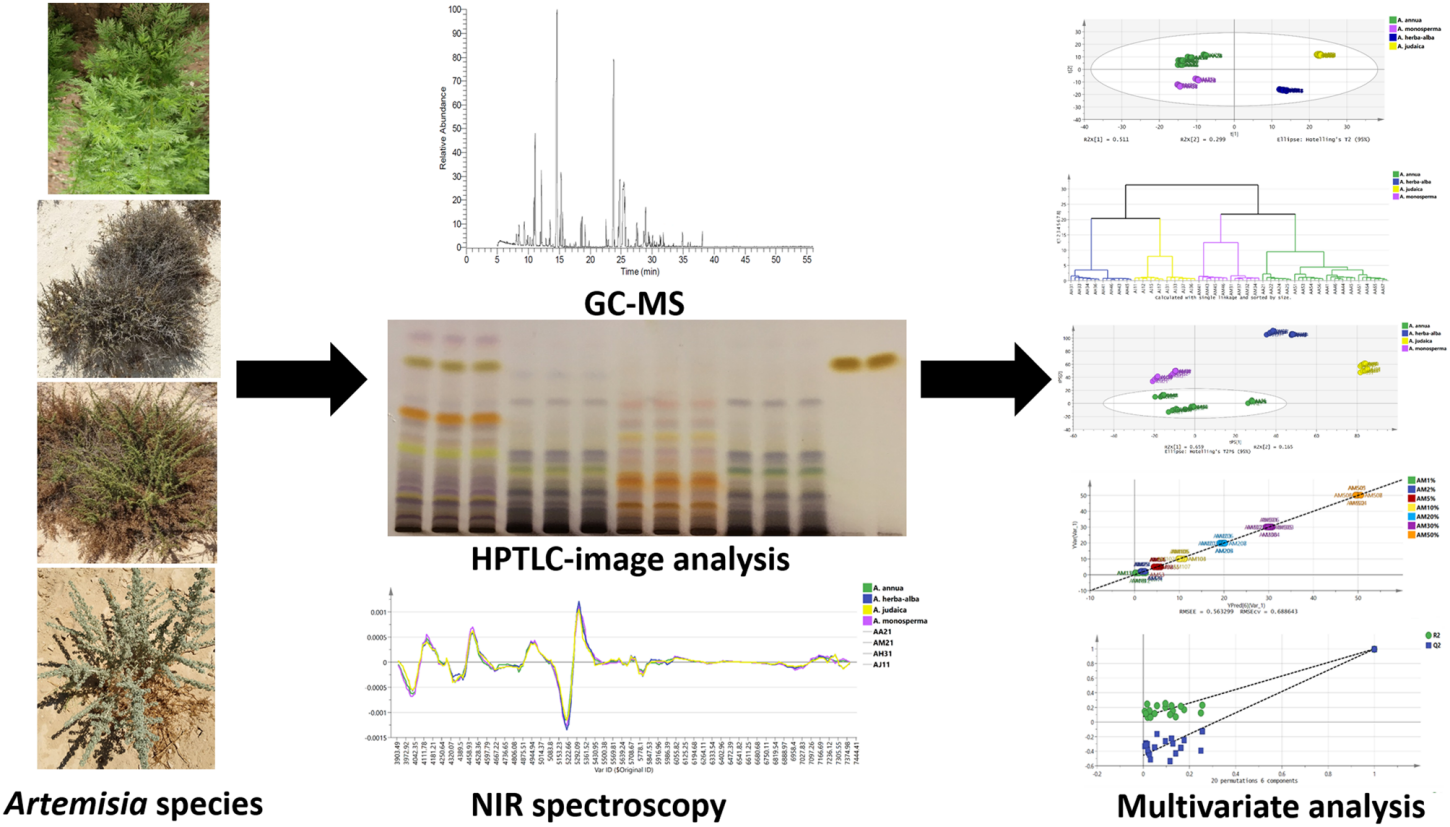

**Supplementary Information:**

The online version contains supplementary material available at 10.1186/s13065-025-01467-5.

## Introduction

Genus *Artemisia* is one of the biggest and most universally distributed genera of family Astraceae (Compositae), constituting over 500 different species, that are spread worldwide in the temperate zones of Europe, Africa, Asia and North America. The genus is known for its diverse phytochemical constituents with a wide range of medicinal effects. Genus *Artemisia* has a long history in traditional medicine and many of its species still have unexplored potential [1]. The most famous of *Artemisia* species is *Artemisia annua* L. (sweet wormwood or qinghao in China), widely used in folk medicine for treatment of chills and fevers. The sesquiterpene lactone, artemisinin, was later isolated from *A. annua* and identified for its antimalarial activity. Artemisinin-based Combination Therapy (ACT) is recommended by the World Health Organization (WHO) as the first line treatment for malaria. In addition, artemisinin garnered interest for its anticancer properties as of the past decade. *A. annua* is renowned worldwide and cultivated in various regions for its artemisinin content. It is especially important in Africa were malaria is prevalent [2, 3]. Among the promising species of genus *Artemisia*, *Artemisia herba-alba* Asso, *Artemisia monosperma* Del. and *Artemisia judaica* L. are wild species growing in Egyptian deserts and sandy Mediterranean regions, and are known for their traditional uses in many cultures [4]. *A. herba-alba*, given the name of desert wormwood in English and Shih in Arabic, is traditionally used as anti-spasmodic, anthelmintic, anti-inflammatory and for wound healing [5, 6], in addition to its antibacterial and antifungal properties [7]. *A. monosperma* is used in folk medicine for gastrointestinal disorders, fever, hypertension and diabetes [8, 9]. Finally, *A. judaica*, “Shih balady” in Arabic, is known as a restorative plant in traditional medicine used to improve vision, enhance immunity and cardiovascular health [10, 11]. Additionally, essential oils of genus *Artemisia* exhibit broad spectrum of activities including antiseptic, fungicidal and antibacterial activities owing to the lipophilic property of their components. They also display analgesic, anti-inflammatory, antispasmodic and local anesthetic effects [12]. The medicinal potential of *Artemisia* species prompted research interest into their chemical profiles.

The aim of this article is the comparative phytochemical profiling of the volatile oils, total extracts and plant powders of the aerial parts of *A. annua*, *A. herba-alba*, *A. monosperma* and *A. judaica* using gas chromatography-mass spectrometry (GC-MS), high-performance thin-layer chromatography (HPTLC) and near infrared (NIR) spectroscopy coupled with multivariate analysis for their classification, identification and authentication. Since *A. annua* is an important industrial crop that is usually cultivated for its artemisinin content, it is essential to ensure its purity and avoid adulteration with other *Artemisia* species. Hence, NIR spectroscopy was also used to differentiate the four species and for quality control of *A. annua*.

## Results and discussion

### Comparative evaluation of the volatile oils of *Artemisia* species

Whitish to yellowish volatile oils with strong pleasant odor were extracted from the aerial parts of *A. annua*, *A. herba-alba*, *A. monosperma* and *A. judaica* with a yield of 0.8%, 1%, 1.12% and 1.2%, respectively. This revealed that *A. judaica* yielded the highest amount of oil followed by *A. monosperma* and *A. herba-alba* then finally *A. annua* with the lowest yield. The qualitative and quantitative composition of the volatile oils were investigated using GC-MS, chromatograms shown in Fig. S1, where a total of 92 compounds were identified; 48 in *A. annua*, 43 in *A. herba-alba*, 44 in *A. monosperma* and 48 in *A. judaica*. The identified compounds are listed in Table 1 by order of their elution on TG-5MS column.

**Table 1:** Chemical profiles of the volatile oils of *A. annua*, *A. herba-alba*, *A. monosperma* and *A. judaica*

No.	Compounds	Rt	RI	A. annua	A. herba-alba	A. monosperma	A. judaica
1	Santolina Triene	7.44	911	**-**	0.53	**-**	**-**
2	α-thujene	8.03	929	0.19	0.15	0.65	0.16
3	α-pinene	8.1	933	0.54	3.27	9.39	0.45
4	Camphene	8.55	947	1.15	9.03	**-**	1.01
5	Thuja-2,4(10)-diene	8.81	954	**-**	0.87	**-**	**-**
6	Sabinene	9.37	972	1.54	4.88	**-**	1.27
7	β-pinene	9.73	984	0.24	0.41	13.95	0.22
8	β-Myrcene	9.9	989	0.46	0.66	5.28	0.37
9	Yomogi Alcohol	10.14	998	0.44	1.92	**-**	0.37
10	α-phellandrene	10.24	1001	**-**	**-**	0.74	**-**
11	δ-3-carene	10.31	1004	**-**	**-**	1.74	0.18
12	α-terpinene	10.69	1015	0.29	2.26	**-**	**-**
13	P-cymene	10.93	1022	2.23	2.55	**-**	1.77
14	trans-2,7-Dimethyl-4,6-octadien-2-ol	11.01	1025	**-**	1.82	**-**	**-**
15	Limonene	11.1	1028	**-**	**-**	5.42	**-**
16	1,8-Cineole (Eucalyptol)	11.19	1031	5.33	3.61	**-**	4.54
17	Cis-β-Ocimene	11.42	1038	**-**	**-**	4	**-**
18	7-Methylbicyclo [2.2.1] hept-5-en-2-one	11.43	1039	**-**	0.7	**-**	**-**
19	Trans-β-Ocimene	11.72	1047	**-**	**-**	3.59	0.4
20	γ-Terpinene	11.98	1056	0.55	1.39	1.21	**-**
21	Artemisia ketone	12.13	1061	3.02	**-**	**-**	2.54
22	Artemisia Alcohol	12.69	1079	0.55	0.51	**-**	0.47
23	α-terpinolene	12.85	1084	**-**	**-**	13.61	**-**
24	Linalool	13.31	1098	0.38	**-**	**-**	0.29
25	α-thujone	13.51	1105	0.86	2.17	**-**	0.61
26	β-thujone	13.77	1113	**-**	0.24	**-**	**-**
27	Chrysanthenone	13.96	1120	**-**	0.52	0.14	**-**
28	Cis-Verbenol	14.25	1129	**-**	0.3	**-**	**-**
29	Trans-p-2-Menthen-1-ol	14.26	1129	**-**	**-**	0.3	**-**
30	Camphor	14.64	1142	26.45	0.49	**-**	23.19
31	Lavandulol	15.25	1162	0.42	0.21	**-**	0.41
32	Borneol	15.31	1163	3.91	0.27	**-**	3.84
33	Terpinen-4-ol	15.62	1173	1.09	0.17	1.55	1.13
34	Cis-Pinocarveol	15.95	1183	**-**	22.6	**-**	**-**
35	α-terpineol	16.21	1191	0.2	0.13	0.17	0.23
36	Myrtenol	16.43	1198	**-**	1.05	**-**	**-**
37	Cis-piperitol	16.5	1200	**-**	4.12	**-**	**-**
38	n-Decanal	17.2	1223	**-**	**-**	0.37	**-**
39	Dihydrocarvone	17.65	1237	0.18	**-**	**-**	**-**
40	Verbenone	18.5	1264	0.66	**-**	**-**	**-**
41	Linalyl acetate	18.58	1267	**-**	**-**	0.21	0.63
42	p-cymen-9-ol	18.67	1270	0.74	**-**	0.29	0.77
43	Thymol	19.1	1284	0.66	**-**	2.27	0.7
44	Trans-Chrysanthenyl acetate	19.61	1300	**-**	13.88	**-**	**-**
45	Cuminaldehyde	19.84	1307	0.19	0.12	**-**	0.16
46	Cis-Chrysanthenyl acetate	20.36	1324	**-**	9.35	0.33	**-**
47	Piperitone	20.45	1327	**-**	**-**	**-**	17.14
48	α-Copaene	22.34	1387	0.83	**-**	**-**	0.9
49	β-Cubebene	22.45	1391	0.22	**-**	**-**	0.26
50	β-Elemene	22.76	1400	0.44	0.77	0.62	0.34
51	Cyperene	22.97	1407	**-**	0.37	**-**	**-**
52	Cis-threo-Davanafuran	23.38	1420	**-**	**-**	1.06	**-**
53	β-Caryophyllene	23.62	1428	17.75	0.33	0.19	**-**
54	Geranyl acetone	24.08	1443	**-**	**-**	0.25	**-**
55	α-Humulene	24.32	1450	0.42	**-**	0.13	0.37
56	Trans-β-Farnesene	24.59	1459	5.72	**-**	**-**	6.87
57	α-curcumene	25.12	1476	**-**	**-**	2.78	**-**
58	Cis-muurola-4(14),5-diene	25.23	1479	**-**	1.15	**-**	**-**
59	Trans-Ethyl cinnamate	25.24	1480	**-**	**-**	**-**	12.65
60	Germacrene D	25.28	1483	9.81	**-**	2.04	**-**
61	α-Selinene	25.39	1487	2.88	**-**	**-**	4.04
62	β-Bisabolene	25.53	1492	**-**	**-**	**-**	0.81
63	γ-Amorphene	25.54	1493	0.63	**-**	**-**	**-**
64	α-muurolene	25.64	1497	**-**	0.48	1.08	**-**
65	δ-Cadinene	25.92	1506	0.21	0.34	0.43	**-**
66	Isoshyobunone	26.32	1518	**-**	**-**	2.68	**-**
67	Calamenene	26.71	1530	**-**	**-**	0.98	**-**
68	α-Calacorene	26.83	1534	**-**	**-**	1.54	**-**
69	Widdrol	26.93	1538	**-**	**-**	1.76	**-**
70	Nerolidol	27.03	1543	**-**	**-**	**-**	0.17
71	Citronellyl isovalerate	27.1	1547	**-**	**-**	1.52	**-**
72	Germacrene D-4-ol	27.3	1551	1.16	**-**	**-**	1.43
73	(-)-Spathulenol	27.5	1558	0.78	0.26	11.71	0.96
74	Davanone	27.95	1573	**-**	**-**	1.08	**-**
75	Caryophyllene oxide	28.37	1584	0.5	2.91	**-**	0.62
76	Carotol	28.61	1593	**-**	**-**	0.13	**-**
77	1-epi-Cubenol	28.74	1599	1.91	**-**	**-**	2.56
78	epi-α-Cadinol	29.05	1611	**-**	**-**	0.15	**-**
79	α-Muurolol	29.21	1617	0.59	**-**	0.33	0.63
80	8-Isopropenyl-1,3,3,7-tetramethyl-bicyclo[5.1.0]-oct-5-en-2-one	29.37	1619	0.6	**-**	**-**	0.67
81	γ-Gurjunene epoxide	29.43	1621	**-**	0.81	**-**	**-**
82	α-Humulene epoxide II	29.5	1625	**-**	**-**	0.72	**-**
83	β-Eudesmol	29.64	1629	**-**	**-**	1.35	**-**
84	α-Cadinol	29.87	1632	0.44	0.14	0.59	0.57
85	β-bisabolol	29.93	1635	**-**	**-**	0.43	**-**
86	Cedr-8-en-13-ol	30.15	1641	0.26	**-**	**-**	0.2
87	9H-Cycloisolongifolene, 8-oxo-	30.5	1653	**-**	**-**	**-**	0.2
88	1-Methylene-2b-hydroxymethyl-3,3-dimethyl-4b-(3-methylbut-2-enyl)-cyclohexane	31.07	1675	0.52	**-**	**-**	0.57
89	Isolongifolol	31.25	1681	0.28	0.17	**-**	0.29
90	β-Santalol	31.65	1693	0.4	0.23	0.13	0.48
91	Auraptenol	34.68	1792	0.8	**-**	**-**	0.95
92	Phytol	37.92	1899	0.4	**-**	**-**	0.4
	**Total identified**			**99.79%**	**98.89%**	**98.14%**	**99.82%**
	**Monoterpene hydrocarbons**			7.19%	26%	59.58%	5.83%
	**Oxgyenated monoterpenes**			45.08%	63.48%	5.18%	57.04%
	**Sesquiterpene hydrocarbons**			38.91%	3.44%	9.79%	13.59%
	**Oxgyenated sesquiterpenes**			7.44%	4.52%	22.58%	9.35%
	**Non-terpenoid compounds**			0.8%	0.7%	1.43%	13.6%
	**Oxgyenated Diterpenes**			0.4%	-	-	0.4%

Rt, retention time (min.); RI, retention index on TG-5MS column relative to n-alkanes (C5-C28); Identification of compounds based on MS and RI by comparison to the NIST, WILEY and ADAMS libraries; Quantity of identified compounds presented as % relative peak area

The identified compounds from the volatile oils of *A. annua*, *A. herba-alba*, *A. monosperma* and *A. judaica* constituted 99.79, 98.89, 98.14 and 99.82% of the total mass, respectively. The classes of the identified compounds were denoted as monoterpenes and sesquiterpenes, including hydrocarbons or oxygenated terpenes, as well as non-terpenoid compounds (Fig. S2). Monoterpenes were the predominant class in the volatile oils of the four species especially *A. herba-alba*. In addition to the high content of monoterpenes, the other three species also revealed high amount of sesquiterpenes. Monoterpenes constituted 89.48% of the chemical profile of *A. herba-alba* oil with 26% monoterpene hydrocarbons and 63.48% oxygenated monoterpenes. On the other hand, the chemical profile of the volatile oil of *A. annua* was mainly composed of 52.27% monoterpenes, of which 45.08% were oxygenated, and 46.35% sesquiterpenes with hydrocarbons representing 38.91%. Monoterpenes were also the prevalent class in *A. monosperma* and *A. judaica* as 64.76% and 62.87%, respectively. However, *A. monosperma* mainly possessed monoterpene hydrocarbons (59.58%) while *A. judaica* volatile oil majorly had oxygenated monoterpenes (57.04%). Both volatile oils also contained 32.37 and 22.94% of sesquiterpenes. In addition, non-terpenoid compounds constituted 13.6% of *A. judaica* oil. Another study on Egyptian *Artemisia* species reported that oxygenated monoterpenes were the significant category in *A. herba-alba* (75.31%) and *A. judaica* (83.07%), whereas monoterpene hydrocarbons were the major category in *A. monosperma* (36.23%) followed by oxygenated sesquiterpenes (37.08%) [13]. This agrees with our findings concerning the three species, however we reported lower content of oxygenated monoterpenes in *A. judaica* with higher amounts of sesquiterpenes and non-terpenoid compounds. The volatile oil of *A. annua* collected from India was characterized by 65.7% monoterpenes and 27.3% sesquiterpenes [14], similarly the oils from the plants cultivated in Italy and Korea were rich in mono- and sesquiterpenes [15, 16]. This is in accordance with our analysis of the volatile oil of *A. annua* cultivated in Egypt.

Among the predominant monoterpenes in *A. herba-alba* oil, camphene (9.03%), sabinene (4.88%), cis-pinocarveol (22.6%), cis-piperitol (4.12%), trans-chrysanthenyl acetate (13.88%) and cis-chrysanthenyl acetate (9.35%) were the major constituents, which slightly differs from previous report of the Egyptian species [13]. Camphene, sabinene, chrysanthenone and chrysanthenyl acetate were previously reported in *A. herba-alba* from Morocco [7, 17], Libya [18], Algeria [19] and Tunisia [20].Trans-pinocarveol was previously reported as a major compound in the volatile oil of Algerian *A. herba-alba* [21] and in lower amount in the Libyan plant [18]. Although camphor and thujones were found in high amounts in the Algerian [21–24], Tunisian [20, 25, 26] and Moroccan [7, 27] plant, they are present in low amount in the Egyptian *A. herba-alba*. Monoterpene hydrocarbons, α-pinene (9.39%), β-pinene (13.95%), β-Myrcene (5.28%), limonene (5.42%) and α-terpinolene (13.61%) along with the oxygenated sesquiterpene (-)-spathulenol (11.71%) constituted the major components of *A. monosperma* oil. This closely matches previous reports of Egyptian and Saudi Arabian plants [13, 28, 29].

Camphor is a major constituent in the volatile oils of both *A. annua* (26.45%) and *A. judaica* (23.19%). In addition to camphor, *A. annua* oil depicted high levels of β-caryophyllene (17.75%), germacrene D (9.81%), trans-β-farnesene (5.72%), 1,8-cineole (5.33%) and artemisia ketone (3.02%). This is consistent with *A. annua* cultivated in Italy [15, 30] and Korea [16] except for the lower levels of artemisia ketone in the Egyptian plant. As for *A. judaica*, along with camphor, piperitone (17.14%), trans-ethyl cinnamate (12.65%) and 1,8-Cineole (4.54%) represented major components of the oil. This corresponds with previous investigations of the volatile oil of *A. judaica* from Egypt [13, 31], Algeria [21, 32], Jordan [33], Saudi Arabia [34] and Libya [18].

The volatile oil compositions of the studied *Artemisia* species mostly correspond with previous investigations in Egypt and other countries. However, variations in the identified major constituents can be detected. These variations might be attributed to environmental conditions (climate and soil), geographical origin, plant age and harvesting season.

**Fig. 1:**
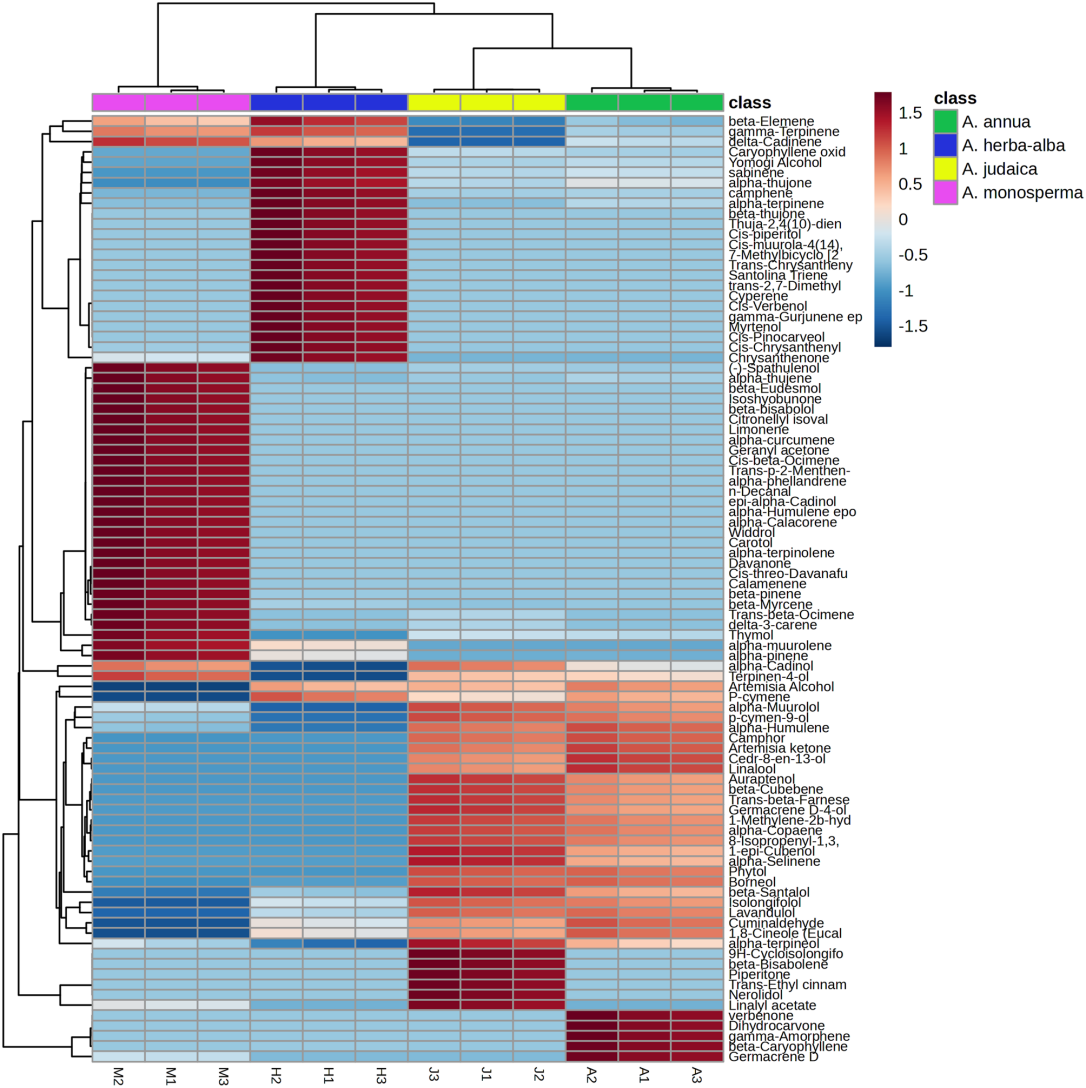
Hierarchical clustering analysis heatmap of PCA model of all identified constituents of volatile oils of the studied *Artemisia* species. Brick red and blue designate higher and lower correlation to the clustering of the species, respectively

GC-MS linked to multivariate analysis was performed to analyze the clustering pattern of the studied *Artemisia* species based on their volatile oil chemical profiles. Unsupervised pattern recognition is a mean to visualize data graphically without direction. It reduces the dimensionality of datasets while maintaining and maximizing variability to discern hidden data patterns and samples’ distribution correlated to their chemical diversity. PCA model was employed for unsupervised pattern recognition. The score scatter plot of the PCA model (Fig. S3) showed a clear grouping of the *Artemisia* samples where PC1 and PC2 variability in the samples constituted 51.7% and 39.2%, respectively with a combined variance of 90.9% and an adjusted ellipse Hoteling at 95%. *A. monosperma* samples were clustered along the positive side of both PC1 and PC2 while *A. herba-alba* samples were gathered along the positive side of PC1 and negative side of PC2. Both *A. annua* and *A. judaica* samples were grouped in the negative side of PC1 and positive side of PC2. The validity of the model was confirmed as R^2^ and Q^2^ (goodness of fit and prediction) were estimated at 0.999 and 0.997, respectively. The scores from PCA were utilized to construct HCA derived dendrogram (Fig. 1) which exposed two main clades. One clade comprised *A. monosperma* while the other clade included the other three species where *A. herba alba* was grouped in one subclade and *A. annua* and *A. judaica* were separated from each other in another subclade. Finally, the HCA heatmap (Fig. 1) was created to distinguish the rationale behind the observed clustering pattern. Several chemical compounds contributed (brick red) to the distinct clustering of the samples of which the major compounds in each volatile oil were main contributors such as α-pinene, β-pinene, β-Myrcene, limonene, α-terpinolene and (-)-spathulenol in *A. monosperma*, camphene, sabinene, cis-pinocarveol, cis-piperitol, trans-chrysanthenyl acetate and cis-chrysanthenyl acetate in *A. herba-alba*, piperitone and trans-ethyl cinnamate in *A. judaica*, β-caryophyllene and germacrene D in *A. annua*.

### Comparative analysis of the total alcoholic extracts of *Artemisia* species

Comparative chemical profiling of total alcoholic extracts of *A. annua*, *A. herba-alba*, *A. monosperma* and *A. judaica* was endeavored using HPTLC-image analysis combined to chemometry tracking certain chemical markers based on literature survey of genus *Artemisia* aiming at authentication and discrimination of the studied *Artemisia* species. Various classes of therapeutically active constituents were previously reported in *Artemisia* species such as sesquiterpene lactones, coumarins, flavonoids and phenolic acids [4, 35–39].

The chemical profiles/fingerprints were evaluated by tracking representative constituents of these chemical classes. The important sesquiterpene lactone, artemisinin, was tracked in plate I and revealed by anisaldehyde/sulphuric acid spray reagent. Coumarins were tracked in the same plate by their fluorescence under UV light at 366 nm while phenolic acids and flavonoids were tracked by their fluorescence quenching under UV light at 254 nm in plate II.

Untargeted pattern recognition technique was accomplished via digitalization of plates I and II. Using PCA score, HCA was attempted as a clustering method for reliable division of dataset yielding HCA dendrograms representing the clustering patterns among samples based on their similarity index; expressed as Euclidean distance and calculated using single linkage algorithm via SIMCA + 14.1 software (Unmetrics AB, Umea, Sweden). The loading plots corresponding to PCA, are generated for tracking R_f_ zones most contributing to variance and hence responsible for the obtained clustering patterns among samples.

**Fig. 2:**
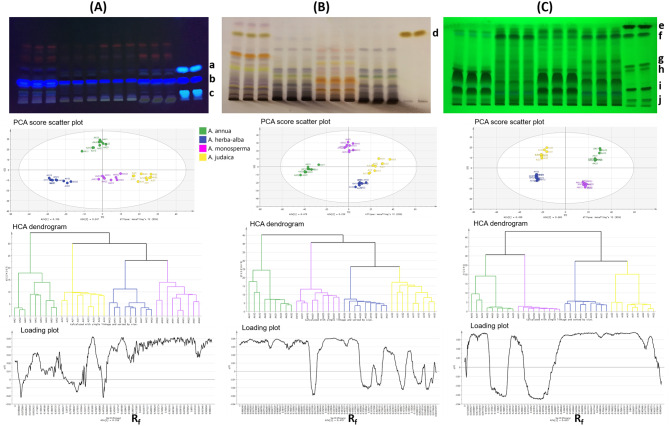
HPTLC plates of representative samples of the studied *Artemisia* species (Tracks 1–3; *A. annua* total extracts, Tracks 4–6; *A. herba-alba* total extracts, Tracks 7–9; *A. monosperma* total extracts, Tracks 10–12; *A. judaica* total extracts) **(A)** Plate I viewed under UV at 366 nm [Tracks 13&14; Ref MIX 1 of umbelliferone (a), scopoletin (b) and aesculetin (c)]. **(B)** Plate I viewed in white light after post-chromatographic derivatization with anisaldehyde/sulphuric acid spray reagent [Tracks 13&14; reference artemisinin standard (d)]. **(C)** Plate II viewed under UV at 254 nm [Tracks 13&14; Ref MIX 2 of kaempferol (e), vitexin (g), quercetin-3-galactoside (h) and rutin (i) & phenolic acids; caffeic (f) and chlorogenic acids (j)] along with their untargeted PCA score scatter plots, HCA dendrograms and corresponding loading plots

Plate I was visualized under UV light at 366 nm for evaluation of coumarins in the tested samples. The digitalized plate I (Fig. 2A) comprising 36 samples X 859 variables was analyzed and subjected to unsupervised pattern recognition method. The resultant PCA score scatter plot of the total extracts of the studied species (Fig. 2A), with PC1 and PC2 representing 60.3% total variance, showed clustering of *A. annua* samples along the positive side of PC2 in the upper half of the Hoteling ellipse apart from the other *Artemisia* species while *A. judaica* samples were collected along the positive side of PC1 and negative side of PC2 with *A. monosperma* clustered almost in the same quarter of the Hoteling ellipse when adjusted at 95% confidence level. Meanwhile, *A. herba-alba* samples were segregated along the negative side of both PC1 and PC2. This was reflected on the HCA dendrogram (Fig. 2A) dividing *Artemisia* species into two separate clades; one for *A. annua* and one comprised the other species in two subclades; one for *A. judaica* and the other gathered *A. monosperma* and *A. herba-alba* separately. The corresponding loading plot (Fig. 2A) revealed zones accounting for the coumarins, aesculetin, scopoletin and umbelliferone, R_f_ of 0.11, 0.24 and 0.39, respectively. Scopoletin is a major distinctive coumarin in *Artemisia* species [40, 41] with *A. annua* approximately showing the highest relative amount followed by *A. judaica*. Aesculetin and umbelliferone can be detected as minor coumarins in all extracts with *A. annua* and *A. judaica* showing relatively more intense spots compared to *A. herba-alba* and *A. monosperma*. Aesculetin was previously reported in *A. annua* and other *Artemisia* species [38, 42, 43] and umbelliferone was detected in the genus [42–45].

Sequentially, Plate I was visualized in white light following post-chromatographic derivatization with anisaldehyde/sulphuric acid reagent (Fig. 2B) for tracking of the valuable sesquiterpene lactone (artemisinin) in the studied *Artemisia* species. PCA score scatter plot (Fig. 2B) of the digitalized plate (36 samples X 1173 variables) revealed that *A. annua* samples were clustered almost along the negative side of both PC1 and PC2 while *A. monosperma* samples were localized along the positive side of PC2 and in between positive and negative sides of PC1. *A. herba-alba* and *A. judaica* were more closely clustered, both samples were gathered along the positive side of PC1 where *A. herba-alba* samples were at the negative side of PC2, while *A. judaica* samples were scattered between the positive and negative sides of PC2. PC1 and PC2 accounted for 47.9% and 25.9% of total variance among samples. The correlated HCA dendrogram (Fig. 2B) showed clustering pattern similar to that exhibited for coumarins content, it separated *A. annua* in one clade while the other species were divided in two separate subclades of the other clade, with *A. judaica* and *A. herba-alba* sharing the same subclade in a distinctive manner. The corresponding loading plot (Fig. 2B) showed zones at R_f_ 0.09, 0.17, 0.29, 0.35, 0.45, 0.64 and 0.84 to be contributors to the clustering pattern of the samples. The zone at R_f_ 0.84 accounted for the sesquiterpene lactone, artemisinin, which can be clearly observed to be present only in the total extract of *A. annua* and undetected in the total extracts of *A. herba-alba*, *A. monosperma* and *A. judaica*. This confirms the discriminatory power of artemisinin in differentiating and distinguishing these *Artemisia* species as artemisinin is a characteristic chemical constituent unique for the *annua* species and widely known for its antimalarial activity.

Regarding plate II visualized under UV light at 254 nm (Fig. 2C) tracking flavonoids and phenolic acids, PC1 and PC2 of the PCA score scatter plot (Fig. 2C) of the digitalized plate (36 samples X 1047 variables) accounted for 66.8% of variance among samples. It revealed that *A. annua* samples were clustered along the positive side of both PC1 and PC2 while *A. herba-alba* samples were clustered along the negative side of both PC1 and PC2. *A. monosperma* samples were localized along the positive side of PC1 and the negative side of PC2. On the other hand, *A. judaica* samples were gathered along the negative side of PC1 and the positive side of PC2. This clustering pattern was relevant in the associated HCA dendrogram (Fig. 2C) that distinctly grouped *A. annua* and *A. monosperma* in one clade and *A. herba-alba* and *A. judaica* in another clade. The corresponding loading plot (Fig. 2C) showed the most contributing zones to the clustering pattern. Several phenolic acids and flavonoids were detected in the extracts. The phenolic acids, chlorogenic acid (R_f_ 0.07) and caffeic acid (R_f_ 0.92), can be ascertained in the four species with caffeic acid present in higher relative abundance in *A. herba-alba* and *A. judaica*. Both phenolic acids were previously detected in *A. annua* and other *Artemisia* species [36, 37, 41]. Kaempferol flavonoid aglycone (R_f_ 0.98) can be detected as a minor compound in all species except *A. annua*. Rutin (R_f_ 0.18) was revealed as a major spot in *A. annua* and *A. monosperma* while the other two species showed minor amount. Rutin is a flavonoid that is usually present in *Artemisia* species [38, 46, 47]. Vitexin (R_f_ 0.42) can be identified in *A. annua* and *A. monosperma*, on the other hand, quercetin-3-galactoside (R_f_ 0.37) can be identified in *A. annua* and *A. judaica*. At the end, HPTLC analysis provided insight into the chemical composition of the alcoholic extracts of the *Artemisia* species in relation to previous reports on the genus.

### Authentication, classification and quality control of *Artemisia* species using NIR spectroscopy

#### NIR spectral analysis of Artemisia species

**Fig. 3:**
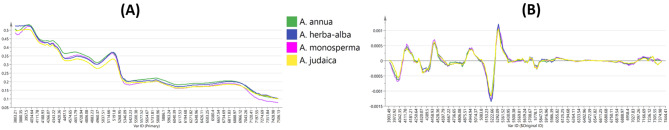
Overlay of **(A)** raw NIR spectra and **(B)** Second derivative derivative, WDS and SGF converted NIR spectra of representative *Artemisia* samples spanning the range of 3800–7500 cm^− 1^

No clear differences can be discerned amongst the raw NIR spectra of *A. annua*, *A. herba-alba*, *A. monosperma* and *A. judaica* within the spectral range of 3800–7500 cm^− 1^ (Fig. 3A). Nevertheless, preprocessing by application of second derivative followed by wavelet denoising (WDS) and Savitzky-Golay filter (SGF) depicted noticeable distinctions in the spectral regions spanning 3900–4200, 4320–4420, 4500–4800, 5100–5500, 7100–7400 cm^− 1^ as shown in Fig. 3B.

The spectral features of the samples (Fig. 3B) comprised signals characteristic for lactones at approximately 5700–5800, 5100–5400, 4700 and 4300 cm^− 1^ representing symmetric and asymmetric CH_2_ stretching overtone bands, carbonyl group second overtone, C-O stretching overtone and C-H bending vibration, respectively [48, 49]. Also, signals indicative of terpenes can be detected correlated to the high content of mono- and sesquiterpenes. In addition to the occurrence of signals at 6020–6100 cm^− 1^ signifying the first overtone of both -COC- and vinyl stretching of coumarins while signals in the region of 4200–4700 cm^− 1^ can correspond to the hydroxyl group of phenolics [50, 51]. Bands at 5840–6090 cm^− 1^ correlated to aromatic rings where band at 5920–5950 cm^− 1^ represented the aromatic first overtone of–CH. Moreover, -OH stretching first overtone of sugar moieties were represented by signals in 6600–7090 cm^− 1^ region while -CH aliphatic first overtone of flavone glycosides sugar were denoted at 5630–5800 cm^− 1^ [52, 53].

#### Unsupervised pattern recognition of Artemisia powders

**Fig. 4:**
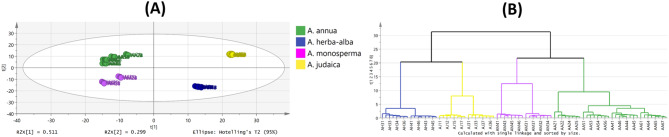
**(A)** PCA score scatter plot and **(B)** HCA dendrogram (single linkage algorithm) of the studied *Artemisia* species powders based on their NIR spectra

The NIR spectral data representing the X-variables were subjected to PCA to explore the grouping pattern of the powders of the studied *Artemisia* species. The score scatter plot of PCA model (Fig. 4A) exposed distinct clustering of the *Artemisia* species with total variance within the samples of 81% and an adjusted ellipse Hoteling at 95% where variability of PC1 and PC2 constituted 51.1% and 29.9%, respectively. *A. annua* samples were clustered along the negative side of PC1 and positive side of PC2 while *A. herba-alba* samples were grouped opposite to it. On the other hand, *A. monosperma* samples were gathered at the negative side of both PC1 and PC2 whereas *A. judaica* samples were clustered at the positive side of both. HCA derived dendrogram based on the PCA model (Fig. 4B) showed two main clades; one comprising *A. annua* and *A. monosperma* in two separate subclades and the other separating *A. herba-alba* and *A. judaica* in two subclades. The corresponding loading plot (Fig. S4) highlighted the regions contributing to the clustering pattern. Supervised class modelling was performed next to consider class membership and authenticate each species.

#### Supervised pattern recognition (SIMCA) of Artemisia powders

**Fig. 5:**
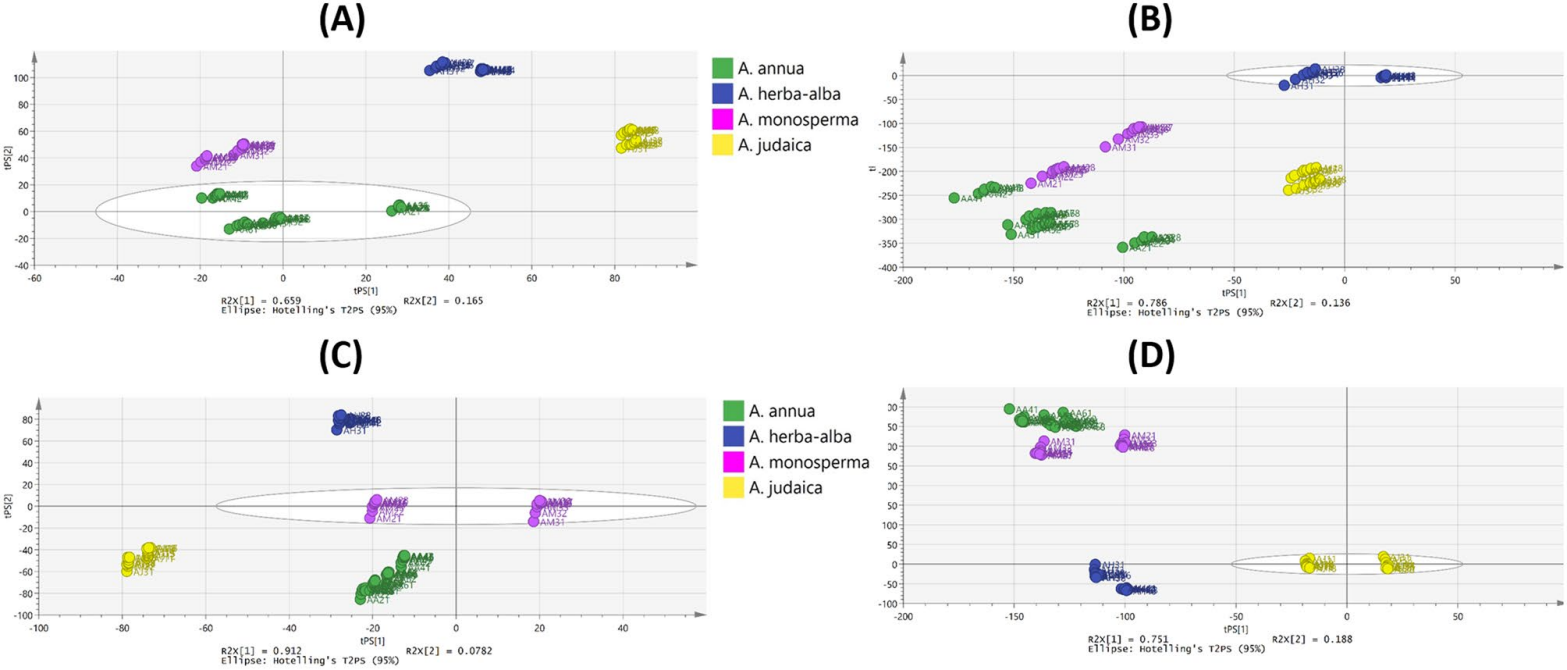
SIMCA score scatter plot of samples applied to *Artemisia* species and constructed using **(A)** *A. annua* model, **(B)** *A. herba-alba* model, **(C)** *A. monosperma* model and **(D)** *A. judaica* model based on the NIR spectra of their powder samples

SIMCA was applied for identification and authentication of the different *Artemisia* species. The SIMCA models were created using NIR data from 3800 to 7500 cm^− 1^ where the data matrix comprised 75 samples of *A. annua*, *A. herba-alba*, *A. monosperma* and *A. judaica* X 481 variables for calibration whereas the test set consisted of 35 samples (Fig. 5). The models were assessed for goodness of fitting and prediction showing R^2^ and Q^2^ above 0.9. Also, specificity, sensitivity, accuracy and efficiency were evaluated (Table 2). Correct classification of the classes assigned to the samples was ensured using Cooman’s plots (Table S1 and Fig. S5) that confirmed the model validity and ability to predict classes while avoiding misclassification.

**Table 2:** Classification parameters for *Artemisia* species using SIMCA model

	Class	NS	Sensitivity	Specificity	Efficacy	Accuracy
Training (calibration) set	*A. annua*	30	96.67%	100%	98.32%	98.67%
	*A. herba-alba*	15	93.33%	100%	96.61%	98.67%
	*A. monosperma*	15	100%	100%	100%	100%
	*A. judaica*	15	93.33%	100%	96.61%	98.67%
Test set	*A. annua*	14	92.86%	100%	96.36%	97.14%
	*A. herba-alba*	7	100%	100%	100%	100%
	*A. monosperma*	7	100%	100%	100%	100%
	*A. judaica*	7	100%	100%	100%	100%

Considering *A. annua* along with the other species (*A. herba-alba*, *A. monosperma* and *A. judaica*), the best SIMCA model was created using 3 principal components where PC1 and PC2 contributed to variance by 65.9% and 16.5%, respectively and the model exhibited sensitivity and specificity of 96.67% and 100%, respectively. Similarly, SIMCA models were constructed for each of *A. herba-alba*, *A. monosperma* and *A. judaica* with the other species using 3 principal components (PC1 and PC2 contribution to variance was 78.6% and 13.6%, 91.2% and 7.82%, 75.1% and 18.8%, respectively) with all models exhibiting high sensitivity and 100% specificity.

The score scatter plots based on the created SIMCA models displayed distinct clustering of each *Artemisia* species with adjusted Hoteling ellipse at 95% which clearly distinguish *A. annua* from the other species (Fig. 5A), *A. herba-alba* from the other species (Fig. 5B), *A. monosperma* from the other species (Fig. 5C) and *A. judaica* from the other species (Fig. 5D). Hence, the models achieved the targeted classification of the species based on the NIR spectra of their powdered samples. The corresponding loading plots (Fig. S6) underscored the NIR regions contributing to the classification of each *Artemisia* species.

#### PLSR models for quality control of A. annua

**Fig. 6:**
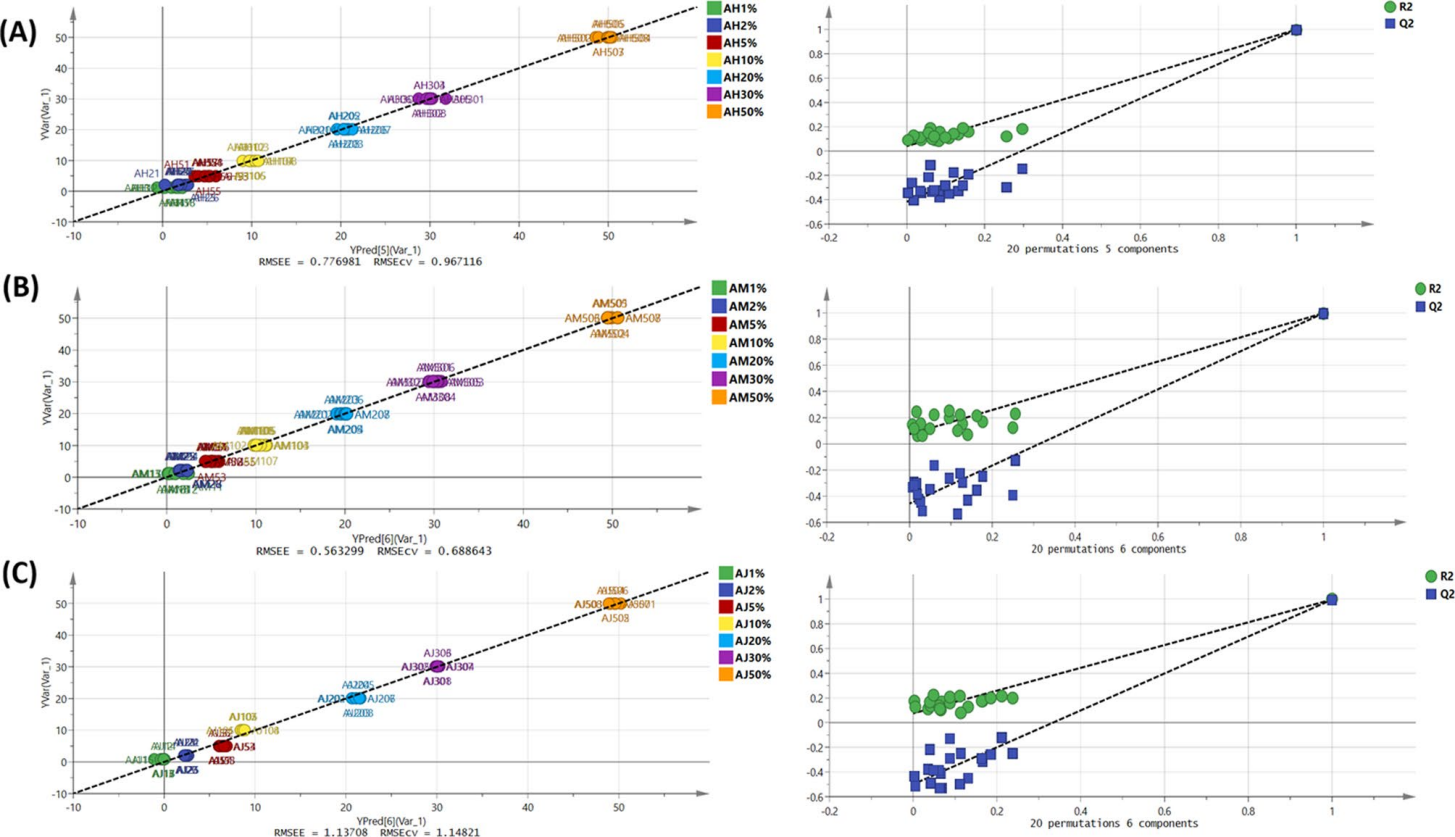
The correlation between observed and predicted values for PLSR model of *A. annua* adulterated with **(A)** *A. herba-alba*, **(B)** *A. monosperma* and **(C)** *A. judaica* along with their permutation plots

Detection of adulteration of *A. annua* is important due to its status as a valuable industrial crop. PLS regression analysis was employed to quantitatively evaluate the occurrence of *Artemisia* species adulterants in *A. annua* powdered samples, considering *A. herba-alba*, *A. monosperma* and *A. judaica*, in levels of adulteration ranging from 1 to 50% (Fig. 6). The X-matrix was represented by NIR spectral variables while the adulteration levels represented the Y-matrix creating a sample X variable arrangement. The calibration set comprised 56 samples, whereas 21 samples of each adulterant were additionally assigned to the test set. The complete multivariate calibration parameters utilized for various adulterants quantitation (Table 3) indicated that the PLSR models created using the calibration set generated commendable outcomes. PLSR models constructed for adulteration prediction showed goodness of fitting and prediction reflected in R^2^ and Q^2^ values. Also, PLSR models were internally and externally validated in terms of the accepted values of root mean square error of calibration (RMSEC), root mean square error of cross validation (RMSECV) and root mean square error of prediction (RMSEP). The calibration performance represented by RMSEC values was in the range of 0.5 to 1.1 for the different adulterants. For comprehensive evaluation, RMSECV values were calculated based on a leave-ten-out cross-validation approach exhibiting a range from 0.6 to 1.1. The RMSECV/RMSEC ratio of the models were below 1.5, indicating a low probability of data overfitting [54]. The robustness of the model fit was confirmed by satisfactory R^2^ values for both the calibration and cross-validation data (Table 3). Residual predictive deviation (RPD) which is calculated as the ratio of standard deviation (SD) of the original data to the RMSEP was used to evaluate the prediction accuracy and robustness of the models. RPD of 2.5-3 demonstrates good prediction accuracy while RPD > 3 reflects excellent prediction accuracy [54–56]. RPD for the models of the three adulterants was higher than 3 (Table 3) indicating that the models are excellent for quality control.

**Table 3:** Multivariate calibration parameters and performance results of PLSR models of cultivated *A. annua* and each of the Egyptian wild *Artemisia* species as adulterants

Adulterants	NS	LV	Regression equation	*R* ^2^	Calibration		Cross validation		Test (Prediction)		RPD	LOD (%)	LOQ (%)
					*R* ^2^	RMSEC	*R* ^2^	RMSECV	*R* ^2^	RMSEP			
*A. herba-alba*	56	5	Y = X − 1.345*10^− 6^	0.9981	0.999	0.776981	0.998	0.967116	0.9976	0.867754	3.11	0.6508–1.2998	1.9525–3.8993
*A. monosperma*	56	6	Y = X + 2.464*10^− 7^	0.999	0.999	0.563299	0.999	0.688643	0.9985	0.660015	3.24	0.7184–1.0825	2.1553–3.2474
*A. judaica*	56	6	Y = X – 2.835*10^− 7^	0.9959	0.999	1.13708	0.995	1.14821	0.9966	0.964232	3.01	0.6902–1.4333	2.0706–4.2998

**Fig. 7:**
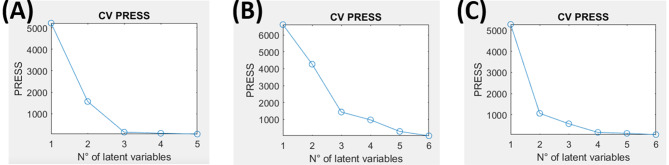
Variations in PRESS as a function of the optimal latent variables number for PLSR model of *A. annua* adulterated with **(A)** *A. herba-alba*, **(B)** *A. monosperma* and **(C)** *A. judaica*

External validation was comprehensively performed, utilizing 21 samples for the test set of each model, and the results were meticulously authenticated through permutation tests. The number of latent variables (LVs) by means of leave-one-out cross-validation are shown in Table 3 and the prediction residual error sum of squares (PRESS) are presented in Fig. 7 which were confirmed by assessment of the permutation plots and its analogous intercept values, as illustrated in Fig. 6. These results represent the suggestion that the models are capable of identifying sample adulterations guaranteeing *A. annua* authenticity excluding data overfitting or noise modelling. Moreover, the ranges for the Limit of Detection (LOD) and Limit of Quantification (LOQ) [minimum to maximum] for the PLSR models are depicted in Table 3. The LOD for all samples was less than 1.5%. The LOQ (less than 4.5%) for *A. herba-alba* was established at 1.95–3.89% while those for *A. monosperma* and *A. judaica* were estimated at 2.15–3.25% and 2.07–4.29%, respectively.

The use of NIR was previously reported for rapid detection of artemisinin in *A. annua* [57, 58]. The described approach of applying NIR for authentication and discrimination of *Artemisia* species unlocks the potential of NIR application beyond detection of specific active constituents and provide a reliable rapid method for quality control of the plant species.

## Experimental

### Plant material

Cultivated aerial parts of *A. annua* L. were collected from the experimental garden of Faculty of Pharmacy, Cairo University, Giza, Egypt on May 2021. Wild aerial parts of *A. herba-alba* Asso and *A. monosperma* Del. were collected from Alexandria northern coast kilo 90 and 83, Egypt, respectively on May 2021. Finally, wild aerial parts of *A. judaica* L. were collected from Red Sea Governorate, Egypt on July 2021. Additional plant samples of the four species were later collected on May 2022. Representative samples were authenticated by Prof. Dr. Amal M. Fakhry (Department of Botany and Microbiology, Faculty of Science, Alexandria University). A voucher specimen of each plant is deposited in Department of Pharmacognosy, Faculty of Pharmacy, Alexandria University, Egypt.

### Profiling *Artemisia* volatile oils by GC-MS

#### Volatile oils extraction

The volatile oils were separately extracted from 250 g of freshly cut aerial parts of each *Artemisia* species by hydrodistillation for 3 h at 50 ℃ using a Clevenger apparatus. The distillate oily layer was separated and dried over anhydrous sodium sulphate. Finally, the extracted volatile oils were stored at 4 ℃ in hermetically sealed glass vials and wrapped in aluminum foil, till GC-MS analysis. The yield of volatile oil extracted from each *Artemisia* species was calculated based on the following equation:

Volatile oil yield (%) = [Volume of oil (ml) / Weight of plant material (g)] x 100.

#### GC-MS analysis of volatile oils

GC-MS analysis was performed for identification and relative quantitation of the extracted volatile oils’ constituents. GC-MS was conducted on a Thermo Scientific, Trace GC Ultra/ISQ Single Quadrupole MS fitted with TG-5MS fused silica capillary column (30 m x 0.25 mm, 0.25 μm film thickness). Electron ionization system with ionization energy of 70 ev and helium carrier gas at a constant flow rate of 1 mL/min were used for GC/MS detection. The temperature of the injector and MS transfer line was set at 280 ℃. The oven temperature program was initially set at 40 ℃ for 3 min then increased to 280 ℃ at a rate of 5 ℃/min and finally held for 5 min. The quantitation of all the identified compounds was indicated as a percent relative peak area and presented as an average of three separate analyses. A tentative identification of the compounds was performed based on the comparison of their relative retention indices on TG-5MS column, calculated relative to a series of n-alkanes (C5-C28), and mass spectra with literature data of the NIST, WILEY and ADAMS libraries.

#### Multivariate statistical analysis

The data obtained from the GC-MS analysis of the four *Artemisia* species, in triplicates, was subjected to unsupervised pattern recognition through principal component analysis (PCA) based on the identified volatile compounds using SIMCA^®^14.1 (Umetrics, Umea, Sweden). In addition, a Hierarchical cluster analysis (HCA) heatmap was constructed using Euclidean distance considering each annotated compound with the aid of Metaboanalyst 5.0 (https://www.metaboanalyst.ca/*).*

### Profiling *Artemisia* total alcoholic extracts by HPTLC-image analysis

#### Preparation of plant extracts samples for HPTLC

Air-dried powdered plant material of each *Artemisia* species (300 g) was macerated in 1 L ethanol (70%) at room temperature for 10 days, then filtered and concentrated under reduced pressure to complete dryness using Buchi Rotavapor R-200, Flawil, Switzerland. 50 mg of the resulting dry residues were precisely weighed and dissolved in 1 ml methanol for subsequent use in HPTLC analysis. All dissolved samples were filtered through an 0.2 μm syringe filter before application on the ready-made HPTLC plates.

#### Preparation of reference standard solutions

Sesquiterpene lactone reference standard: artemisinin, coumarin reference standards: umbelliferone, scopoletin and aesculetin, phenolic acids reference standards: caffeic acid and chlorogenic acid, along with flavonoid reference standards: kaempferol, vitexin, quercetin-3-galactoside and rutin (Sigma-Aldrich, USA) were individually prepared in concentration of 0.5 mg/ml. Reference solution mixture 1 (Ref MIX 1) was prepared by admixing the individual reference standard solutions of umbelliferone, scopoletin and aesculetin. Reference solution mixture 2 (Ref MIX 2) was prepared by admixing the individual reference standard solutions of kaempferol, vitexin, quercetin-3-galactoside and rutin, in addition to caffeic and chlorogenic acids.

#### High performance thin-layer chromatography investigation

10 µl of the total alcoholic extracts of *A. annua*, *A. herba-alba*, *A. monosperma* and *A. judaica* (in triplicate) along with 4 µl of Ref MIX 1 together with 4 µl of artemisinin were applied on plate I. While, plate II comprised 10 µl the total alcoholic extracts of *A. annua*, *A. herba-alba*, *A. monosperma* and *A. judaica* (in triplicate) along with 6 µl of Ref MIX 2.

Samples and Ref MIXs were applied on the HPTLC plates (10 × 20 cm) using a 100 µl syringe by means of CAMAG Linomat V automated spray-on band applicator (Muttenz, Switzerland) managed by WinCats manager software (Camag, 2008). The application settings were adjusted at 15 mm from both the margins and the bottom of the HPTLC plates with bandwidth of 8 mm and inter-band spaces of 5 mm. Each plate was comprised of 14 tracks; 12 tracks for the total extract samples of the four *Artemisia* species and 2 tracks for the reference standards. Different mobile phases were assessed for development of the sample spots with optimal resolution. System I (toluene: ethyl acetate 7.5: 2.5 v/v) was chosen as the optimum developing system for plate I and system II (ethyl acetate: methanol: water: glacial acetic acid 60: 5: 4: 0.25 v/v/v/v) was utilized for plate II. The plates were developed vertically for a distance of 90 mm in twin-trough CAMAG glass chamber (10 × 20 cm) containing 50 ml mobile solvent system. Plate I was visualized under UV light at 366 nm followed by spraying with anisaldehyde/sulphuric acid reagent and heating at 120 ℃ while plate II was visualized under UV light at 254 nm.

#### Image processing and multivariate data analysis

The resulting images of the plates comprising total extracts of *A. annua*, *A. herba-alba*, *A. monosperma* and *A. judaica* developed in each of systems I & II were adjusted via Adobe Photoshop^®^ then processed using ImageJ 1.51h (Wayne Rasband, NIH, USA) for multivariate image analysis providing profile plots for each sample. The profile plots are denoted in two-dimensional plots of pixels’ intensities against their distance along a fixed line. The data matrices assembled using these profile plots were subjected to multivariate data analysis using SIMCA 14.1 software (Unmetrics AB, Umea, Sweden). PCA and HCA were employed for untargeted chemical profiling to decrease the HPTLC profiles’ data dimensionality and explore possible clustering patterns of the studied samples.

### Authentication, differentiation and quality control of *Artemisia* powders by NIR spectroscopy

#### Preparation of samples

44 samples of *A. annua*, 22 samples of each of *A. herba-alba*, *A. monosperma* and *A. judaica* aerial parts were collected in an intact form with a total of 110 samples. Details of the times and locations of samples collection is presented in Table S2. Samples were milled into powder with fine particle size in a Moulinex electric grinder then passed over a 2 mm mesh screen sieve to guarantee particle size uniformity. One gram of each sample was weighed then the samples were allocated into training and test sets randomly. 30 samples of *A. annua* and 15 samples of each of *A. herba-alba*, *A. monosperma* and *A. judaica* constituted the training set, while a test set of 14 *A. annua* samples, 7 samples of each of *A. herba-alba*, *A. monosperma* and *A. judaica* was established.

#### Preparation of adulterated mixtures

*A. annua* samples were methodically comminuted with each of *A. herba-alba*, *A. monosperma* and *A. judaica* for intentional adulteration with adulterant percentages in the range of 1 to 50% (1, 2, 5, 10, 20, 30 and 50%) producing a total of 231 intentionally adulterated mixtures. The binary mixtures with adulterants were randomly assigned to calibration (168 samples) and test (63 samples) sets. Further NIR analyses were performed by using one gram of each mixture and PLSR regression models were assembled from the resulting spectra.

#### NIR spectroscopy for measurements and data acquisition

A multi-purpose analyzer (MPA) FT-NIR spectrometer (Bruker Optics GmbH, Rudolf Plank, Ettlingen, Germany) integrated with an InGaAs (indium-gallium-arsenide) detector was employed for NIR scanning of all samples, in uniform glass vials of 20 mm diameter and height, through sphere module in the wave number range from 3500 to 12,000 cm^− 1^. The NIR spectra were gathered using OPUS spectral acquisition software (version 6.5, Bruker Optics Inc., Germany) in a diffuse reflectance mode with an average of 32 scans at a 16 cm^− 1^ resolution per spectrum, using the powdered samples without prior treatment at room temperature. For each sample, three scans were acquired preceding data analysis then, the average mean-centered spectra were incorporated in our models. Scatter and baseline drift correction was accomplished by executing weighted multiple scatter correction (WMSC) through OPUS software (version 6.5 Bruker Optics Inc.).

#### Pre-treatment of spectral data

Preprocessing of raw NIR spectra of genuine samples and purposely adulterated mixtures was performed via second order derivative, wavelet denoising (WDS) and Savitzky-Golay filter (SGF). SIMCA-P version 14.1 software (Umetrics AB, Umea, Sweden) was employed for preprocessing of spectra and multivariate data analysis.

#### Principal component analysis (PCA) and hierarchical cluster analysis (HCA)

Analysis of the data using PCA can be applied by inspecting the differences in the NIR spectra without any reference to other analytical measurements. The NIR absorbance spectra obtained along the range of 3800–7500 cm^− 1^ were analyzed by PCA to discern outliers, likely patterns and the distribution and diversity of samples correlated to their chemical variability. HCA dendrogram using PCA scores was created by SIMCA-*P* 14.1 software (Umetrics AB, Umea, Sweden) to exhibit the samples grouping patterns based on similarity.

#### Soft independent modelling of class analogy (SIMCA) of the Artemisia species

SIMCA is a supervised categorizing method based on the samples’ similarity associated to preset categories. It is applied for authentication and classification of *A. annua*, *A. herba-alba*, *A. monosperma* and *A. judaica*. SIMCA models were implemented and assessed for goodness of fit and prediction (R^2^ and Q^2^), in addition to, evaluation of classification performance through calculating accuracy, sensitivity, specificity and efficiency. The Coomans’ plot can be used to assess adequate class separation [59–61].

#### Partial least-squares regression (PLSR) prediction model for measuring adulteration content

PLSR models for adulteration of *A. annua* with each of *A. herba-alba*, *A. monosperma* and *A. judaica* were established. The number of latent variables (LVs) in each PLSR model were estimated via Leave-One-Out cross-validation, using the prediction residual error sum of squares (PRESS). PLSR models of the quantity of adulterants were created using Simca-P version 14.1 software (Umetrics AB, Umea, Sweden) for prediction of adulterants’ presence in the mixtures. Three PLSR models were individually implemented for the three *Artemisia* species adulterating *A. annua* by means of PLS-1 where the X-matrix constituted the NIR absorbance values, while the Y-matrix was composed of the percentage of adulterants. Evaluation of the models through R^2^ was carried out. Moreover, RMSEC, RMSECV, RMSEP were evaluated for calibration, cross validation and prediction. RPD (SD/RMSEP) was calculated to assess the predictive ability of the models. Permutations plots were employed to ensure the absence of data over-fit (noise modelling). Additionally, the Limit of Detection (LOD) and Limit of Quantification (LOQ) for the PLSR models were calculated based on the interval approach suggested by Allegrini and Olivieri [62] where they suggested a minimum to maximum range of LOD and LOQ values instead of a single value, designed specifically for multivariate models [63]. The LOD and LOQ were estimated through MATLAB R2017b with the freely available software MVC1 from Instituto de Química del Rosario (IQUIR), which can be opened at https://www.mathworks.com/matlabcentral/fileexchange/134327-first-order-multivariate-calibration-gui [49, 64–67].

## Conclusion

This study offers a comprehensive comparative integrated approach for phytochemical profiling, classification, authentication and quality control of *Artemisia* species, wild and cultivated in Egypt. *Artemisia* species present interesting and valuable industrial crops especially *A. annua* that is cultivated worldwide, additionally the wild species *A. herba-alba*, *A. monosperma* and *A. judaica* possess great medicinal potential and commercial applications. GC-MS investigation of the volatile oil of the four studied species revealed that they are rich in volatile oil components with similarities and variations in their composition. This provided a chemical profile of the volatile oil of each species allowing for their differentiation and fingerprinting. HPTLC-image analysis of their total alcoholic extracts facilitated fast comparative fingerprint profiling of the species revealing their clustering pattern and significant chemical markers including coumarins, the sesquiterpene lactone artemisinin, phenolic acids and flavonoids. Finally, NIR spectroscopy was employed for rapid classification and authentication of the plants’ powdered samples where unsupervised pattern recognition exhibited clear sample grouping followed by SIMCA class modelling for supervised pattern recognition demonstrating high sensitivity, specificity and classification accuracy. Also, quality control of *A. annua* powder was achieved by application of PLSR models to detect adulteration with the other *Artemisia* species. This comprehensive approach utilized complementary chemical profiling methods to draw a complete picture of the fingerprint of each of the studied *Artemisia* species allowing their discrimination and authentication in different forms, in addition to unlocking their future potential. It also highlighted the similarities and diversities of their chemical profiles in relation to previous reports on the studied species and the genus in general.

## Electronic supplementary material

Below is the link to the electronic supplementary material.

Supplementary Material 1

## Data Availability

The data supporting this article have been included in the manuscript and supplementary information. Additional data will be made available upon reasonable request to the corresponding author.

## References

[1] Bora KS, Sharma A. The genus Artemisia: A comprehensive review. Pharm Biol. 2011;49:101–9. 20681755 10.3109/13880209.2010.497815

[2] Soni R, Shankar G, Mukhopadhyay P, Gupta V. A concise review on Artemisia annua L.: A major source of diverse medicinal compounds. Ind Crops Prod. 2022;184:115072.

[3] Brown GD. The biosynthesis of Artemisinin (Qinghaosu) and the phytochemistry of Artemisia annua L. (Qinghao). Molecules. 2010;15:7603–98. 21030913 10.3390/molecules15117603 PMC6259225

[4] Nigam M, Atanassova M, Mishra AP, Pezzani R, Devkota HP, Plygun S, et al. Bioactive compounds and health benefits of Artemisia species. Nat Prod Commun. 2019;14:1934578X19850354.

[5] Mohamed AE-HH, El-Sayed MA, Hegazy ME, Helaly SE, Esmail AM, Mohamed NS. Chemical constituents and biological activities of Artemisia herba-alba. Rec Nat Prod. 2010;4:1–25.

[6] Moufid A, Eddouks M. Artemisia herbaAlba: a popular plant with potential medicinal properties. Pakistan J Biol Sci PJBS. 2012;15:1152–9. 10.3923/pjbs.2012.1152.1159 23755405

[7] Houti H, Ghanmi M, Satrani B, Mansouri FE, Cacciola F, Sadiki M et al. Moroccan endemic *Artemisia herba-alba *essential oil: GC-MS analysis and antibacterial and antifungal investigation. Separations. 2023;10:59.

[8] Qwaider NG, Badawy AM, Ahmed SA, Donia MS. Review Article on chemical constituents and biological activity of Artemisia monosperma. Rec Pharm Biomed Sci. 2023;7:8–12.

[9] Abu-Niaaj LF, Katampe I, Abdulla S. The Pharmacological Properties of Artemisia monosperma (Del.). FASEB J. 2019;33:672.10-672.10.

[10] Moharram FA, Nagy MM, El Dib RA, el-Tantawy MM, El Hossary GG, El-Hosari DG. Pharmacological activity and flavonoids constituents of Artemisia judaica L aerial parts. J Ethnopharmacol. 2021;270:113777. 33412247 10.1016/j.jep.2021.113777

[11] Awad BM, Goda MS, Eltamany EE, Ibrahim AK, Badr JM. Chemistry and biological activities of Artemisia judaica: A mini review. Rec Pharm Biomed Sci. 2022;6:29–49.

[12] Abad MJ, Bedoya LM, Apaza L, Bermejo P. The Artemisia L. Genus: A review of bioactive essential oils. Molecules. 2012;17:2542–66. 22388966 10.3390/molecules17032542 PMC6268508

[13] Amin SM, Hassan HM, El Gendy AE-NG, El-Beih AA, Mohamed TA, Elshamy AI, et al. Comparative chemical study and antimicrobial activity of essential oils of three Artemisia species from Egypt and Saudi Arabia. Flavour Fragr J. 2019;34:450–9.

[14] Mukhtar HM, Ansari SH, Ali M, Mir SR, Abdin MZ, Singh P. GC-MS analysis of volatile oil of aerial parts of Artemisia annua Linn. J Essent Oil Bear Plants. 2007;10:168–71.

[15] Bilia AR, Santomauro F, Sacco C, Bergonzi MC, Donato R. Essential oil of Artemisia annua L.: an extraordinary component with numerous antimicrobial properties. Evid Based Complement Alternat Med. 2014;2014:159819. 24799936 10.1155/2014/159819 PMC3995097

[16] Hong M, Kim M, Jang H, Bo S, Deepa P, Sowndhararajan K, et al. Multivariate analysis of essential oil composition of Artemisia annua L. Collected from different locations in Korea. Molecules. 2023;28:1131. 36770797 10.3390/molecules28031131 PMC9920137

[17] Messaoudi Moussii I, Nayme K, Timinouni M, Jamaleddine J, Filali H, Hakkou F. Synergistic antibacterial effects of Moroccan Artemisia herba Alba, Lavandula angustifolia and Rosmarinus officinalis essential oils. Synergy. 2020;10:100057.

[18] Janaćković P, Novaković J, Soković M, Vujisić L, Giweli AA, Dajić-Stevanović Z, et al. Composition and antimicrobial activity of essential oils of Artemisia Judaica, A. herba-alba and A. arborescens from Libya. Arch Biol Sci. 2015;67:455–66.

[19] Dahmani-Hamzani N, Baaliouamer A. Chemical composition of the Algerian essential oil of Artemisia herba-alba native to Djelfa. Riv. Ital. EPPOS. 2005; 40:7–13.

[20] Eljazi JS, Zarroug Y, Aouini J, Salem N, Bachrouch O, Boushih E, et al. Insecticidal activity of Artemisia herba-alba and effects on wheat flour quality in storage. J Plant Dis Prot. 2020;127:323–33.

[21] Dob T, Benabdelkader T. Chemical composition of the essential oil of Artemisia herba-alba Asso grown in Algeria. J Essent Oil Res. 2006;18:685–90.

[22] Bertella A, Benlahcen K, Abouamama S, Pinto DCGA, Maamar K, Kihal M, et al. Artemisia herba-alba Asso. Essential oil antibacterial activity and acute toxicity. Ind Crops Prod. 2018;116:137–43.

[23] Belhattab R, Amor L, Barroso JG, Pedro LG, Cristina Figueiredo A. Essential oil from Artemisia herba-alba Asso grown wild in Algeria: variability assessment and comparison with an updated literature survey. Arab J Chem. 2014;7:243–51.

[24] Zouaoui N, Chenchouni H, Bouguerra A, Massouras T, Barkat M. Characterization of volatile organic compounds from six aromatic and medicinal plant species growing wild in North African drylands. NFS J. 2020;18:19–28.

[25] Mohsen H, Ali F. Essential oil composition of Artemisia herba-alba from Southern Tunisia. Molecules. 2009;14:1585–94. 19384287 10.3390/molecules14041585 PMC6254365

[26] Younsi F, Trimech R, Boulila A, Ezzine O, Dhahri S, Boussaid M, et al. Essential oil and phenolic compounds of Artemisia herba-alba (Asso.): composition, antioxidant, antiacetylcholinesterase, and antibacterial activities. Int J Food Prop. 2016;19:1425–38.

[27] Paolini J, El Ouariachi EM, Bouyanzer A, Hammouti B, Desjobert J-M, Costa J, et al. Chemical variability of Artemisia herba-alba Asso essential oils from East Morocco. Chem Pap. 2010;64:550–6.

[28] Romeilah RM, El-Beltagi HS, Shalaby EA, Younes KM, El Moll H, Rajendrasozhan S, et al. Antioxidant and cytotoxic activities of Artemisia monosperma L. and tamarix aphylla L. essential oils. Not Bot Horti Agrobot Cluj-Napoca. 2021;49:12233.

[29] Khan M, Mousa AA, Syamasundar KV, Alkhathlan HZ. Determination of chemical constituents of leaf and stem essential oils of Artemisia monosperma from central Saudi Arabia. Nat Prod Commun. 2012;7:1079–82. 22978234

[30] Risaliti L, Pini G, Ascrizzi R, Donato R, Sacco C, Bergonzi MC, et al. Artemisia annua essential oil extraction, characterization, and incorporation in nanoliposomes, smart drug delivery systems against Candida species. J Drug Deliv Sci Technol. 2020;59:101849.

[31] Elshamy A, Abd-ElGawad A, Mohamed T, El Gendy AE-N, Abd El Aty AA, Saleh I, et al. Extraction development for antimicrobial and phytotoxic essential oils from Asteraceae species: Achillea fragrantissima, Artemisia judaica and tanacetum Sinaicum. Flavour Fragr J. 2021;36:352–64.

[32] Zeragui B, Hachem K, Halla N, Kahloula K. Essential oil from Artemisia judaica L. (ssp. sahariensis) flowers as a natural cosmetic preservative: chemical composition, and antioxidant and antibacterial activities. J Essent Oil Bear Plants. 2019;22:685–94.

[33] Abu-Darwish MS, Cabral C, Gonçalves MJ, Cavaleiro C, Cruz MT, Zulfiqar A, et al. Chemical composition and biological activities of Artemisia judaica essential oil from Southern desert of Jordan. J Ethnopharmacol. 2016;191:161–8. 27318275 10.1016/j.jep.2016.06.023

[34] Mohammed HA, Qureshi KA, Ali HM, Al-Omar MS, Khan O, Mohammed SAA. Bio-Evaluation of the wound healing activity of Artemisia judaica L. as part of the plant’s use in traditional medicine; phytochemical, antioxidant, Anti-Inflammatory, and antibiofilm properties of the plant’s essential oils. Antioxidants. 2022;11:332. 35204215 10.3390/antiox11020332 PMC8868479

[35] Ickovski JD, Arsić BB, Mitić MN, Stojković MB, Đorđević MM, Stojanović GS. Chemometric approach to the composition of flavonoid compounds and phenolic acids and antioxidant potential of Artemisia species from different habitats. Chem Biodivers. 2022;19:e202200365. 36315629 10.1002/cbdv.202200365

[36] Nikolova M, Gussev C, Nguyen T. Evaluation of the antioxidant action and flavonoid composition of Artemisia species extracts. Biotechnol Biotechnol Equip. 2010;24:101–3.

[37] Carvalho IS, Cavaco T, Brodelius M. Phenolic composition and antioxidant capacity of six Artemisia species. Ind Crops Prod. 2011;33:382–8.

[38] Ferreira JFS, Luthria DL, Sasaki T, Heyerick A. Flavonoids from Artemisia annua L. as antioxidants and their potential synergism with Artemisinin against malaria and cancer. Molecules. 2010;15:3135–70. 20657468 10.3390/molecules15053135 PMC6263261

[39] Bourgou S, Bettaieb Rebey I, Mkadmini K, Isoda H, Ksouri R, Ksouri WM. LC-ESI-TOF-MS and GC-MS profiling of Artemisia herba-alba and evaluation of its bioactive properties. Food Res Int. 2017;99:702–12. 28784534 10.1016/j.foodres.2017.06.009

[40] Zarrelli A, Pollio A, Aceto S, Romanucci V, Carella F, Stefani P, et al. Optimisation of Artemisinin and Scopoletin extraction from Artemisia annua with a new modern pressurised Cyclic solid-liquid (PCSL) extraction technique. Phytochem Anal. 2019;30:564–71. 31238388 10.1002/pca.2853

[41] Qiu F, Wu S, Lu X, Zhang C, Li J, Gong M, et al. Quality evaluation of the Artemisinin-producing plant Artemisia annua L. based on simultaneous quantification of Artemisinin and six synergistic components and hierarchical cluster analysis. Ind Crops Prod. 2018;118:131–41.

[42] Jung HA, Islam MDN, Kwon YS, Jin SE, Son YK, Park JJ, et al. Extraction and identification of three major aldose reductase inhibitors from Artemisia Montana. Food Chem Toxicol. 2011;49:376–84. 21092751 10.1016/j.fct.2010.11.012

[43] Nurul Islam M, Jung HA, Sohn HS, Kim HM, Choi JS. Potent α-glucosidase and protein tyrosine phosphatase 1B inhibitors from Artemisia capillaris. Arch Pharm Res. 2013;36:542–52. 23435948 10.1007/s12272-013-0069-7

[44] Mamatova AS, Korona-Glowniak I, Skalicka-Woźniak K, Józefczyk A, Wojtanowski KK, Baj T, et al. Phytochemical composition of Wormwood (Artemisia gmelinii) extracts in respect of their antimicrobial activity. BMC Complement Altern Med. 2019;19:288. 31660943 10.1186/s12906-019-2719-x PMC6819330

[45] Cheng F-J, Ho C-Y, Li T-S, Chen Y, Yeh Y-L, Wei Y-L, et al. Umbelliferone and eriodictyol suppress the cellular entry of SARS-CoV-2. Cell Biosci. 2023;13:118. 37381062 10.1186/s13578-023-01070-y PMC10304356

[46] Singh P, Bajpai V, Khandelwal N, Varshney S, Gaikwad AN, Srivastava M, et al. Determination of bioactive compounds of Artemisia spp. Plant extracts by LC–MS/MS technique and their in-vitro anti-adipogenic activity screening. J Pharm Biomed Anal. 2021;193:113707. 33160219 10.1016/j.jpba.2020.113707

[47] Fu C, Yu P, Wang M, Qiu F. Phytochemical analysis and geographic assessment of flavonoids, coumarins and sesquiterpenes in Artemisia annua L. based on HPLC-DAD quantification and LC-ESI-QTOF-MS/MS confirmation. Food Chem. 2020;312:126070. 31911352 10.1016/j.foodchem.2019.126070

[48] Westad F, Schmidt A, Kermit M. Incorporating chemical Band-Assignment in near infrared spectroscopy regression models. J Near Infrared Spectrosc. 2008;16:265–73.

[49] Mahgoub YA, Shawky E, Darwish FA, El Sebakhy NA, El-Hawiet AM. Near-infrared spectroscopy combined with chemometrics for quality control of German chamomile (Matricaria recutita L.) and detection of its adulteration by related toxic plants. Microchem J. 2020;158:105153.

[50] Adu-Amankwa B, Sekyere D, Darkwa NA. Rapid prediction of extractives and polyphenolic contents in Pinus Caribaea bark using near infrared reflectance spectroscopy. Int J Appl. 2011;2:1.

[51] Beć KB, Grabska J, Huck CW. Near-Infrared spectroscopy in Bio-Applications. Molecules. 2020;25:2948. 32604876 10.3390/molecules25122948 PMC7357077

[52] Xue Y, Zhang H, Yang X, Niu L, Zhang X, Liu D, et al. Rapid determination of total flavonoids in a medicinal plant epimedium by Near-infrared reflectance spectroscopy. Chin Bull Bot. 2013;48:65–71.

[53] Shi J, Zou X, Zhao J, Mel H, Wang K, Wang X, et al. Determination of total flavonoids content in fresh Ginkgo Biloba leaf with different colors using near infrared spectroscopy. Spectrochim Acta Part Mol Biomol Spectrosc. 2012;94:271–6. 10.1016/j.saa.2012.03.078 22522302

[54] Birenboim M, Kengisbuch D, Chalupowicz D, Maurer D, Barel S, Chen Y, et al. Use of near-infrared spectroscopy for the classification of medicinal cannabis cultivars and the prediction of their cannabinoid and terpene contents. Phytochemistry. 2022;204:113445. 36165867 10.1016/j.phytochem.2022.113445

[55] Sánchez-Carnerero Callado C, Núñez-Sánchez N, Casano S, Ferreiro-Vera C. The potential of near infrared spectroscopy to estimate the content of cannabinoids in cannabis sativa L.: A comparative study. Talanta. 2018;190:147–57. 30172491 10.1016/j.talanta.2018.07.085

[56] Fan X, Tang S, Li G, Zhou X. Non-Invasive detection of protein content in several types of plant feed materials using a hybrid near infrared spectroscopy model. PLoS ONE. 2016;11:e0163145. 27669013 10.1371/journal.pone.0163145 PMC5036844

[57] Dowell FE, Wang D, Wu X, Dowell KM. Detecting the antimalarial artemisinin in plant extracts using near-infrared spectroscopy. 2014.

[58] Camps C, Toussirot M, Quennoz M, Simonnet X. Determination of Artemisinin and moisture content of Artemisia annua L. Dry powder using a Hand-Held near infrared spectroscopy device. J Near Infrared Spectrosc. 2011;19:191–8.

[59] Giraudo A, Grassi S, Savorani F, Gavoci G, Casiraghi E, Geobaldo F. Determination of the geographical origin of green coffee beans using NIR spectroscopy and multivariate data analysis. Food Control. 2019;99:137–45.

[60] Pérez-Beltrán CH, Zúñiga-Arroyo VM, Andrade JM, Cuadros-Rodríguez L, Pérez-Caballero G, Jiménez-Carvelo AM. A sensor-based methodology to differentiate pure and mixed white tequilas based on fused infrared spectra and multivariate data treatment. Chemosensors. 2021;9:1–13.

[61] Baratloo A, Hosseini M, Negida A, El Ashal G. Part 1: simple definition and calculation of accuracy, sensitivity and specificity. Emerg (Tehran Iran). 2015;3:48–9. PMC4614595 26495380

[62] Allegrini F, Olivieri AC. IUPAC-consistent approach to the limit of detection in partial least-squares calibration. Anal Chem. 2014;86:7858–66. 25008998 10.1021/ac501786u

[63] Olivieri AC. Practical guidelines for reporting results in single- and multi-component analytical calibration: A tutorial. Anal Chim Acta. 2015;868:10–22. 25813230 10.1016/j.aca.2015.01.017

[64] Elfiky AM, Shawky E, Khattab AR, Ibrahim RS. Integration of NIR spectroscopy and chemometrics for authentication and quantitation of adulteration in sweet marjoram (Origanum Majorana L). Microchem J. 2022;183:108125.

[65] Shawky E, Selim DA. Rapid authentication and quality evaluation of cinnamomum verum powder using near-infrared spectroscopy and multivariate analyses. Planta Med. 2018;84:1380–7. 30068001 10.1055/a-0654-5468

[66] Selim DA, Darwish RS, Shawky E. Authentication and detection of common adulterants in clove buds (Syzygium aromaticum L.) powders and oils using near IR combined to multivariate analysis. Microchem J. 2023;191:108890.

[67] Chiappini FA, Goicoechea HC, Olivieri AC. MVC1_GUI: A MATLAB graphical user interface for first-order multivariate calibration. An upgrade including artificial neural networks modelling. Chemom Intell Lab Syst. 2020;206:104162.

