# Supplementary material for: Comparative phytochemical profiling and authentication of four Artemisia species using integrated GC-MS, HPTLC and NIR spectroscopy approach
Source: BMC Chem. 2025 Apr 16;19(1):100. doi: 10.1186/s13065-025-01467-5 (PMC12004654; doi:10.1186/s13065-025-01467-5)
Supplement: Supplementary file 1 — Supplementary Material 1 [file 13065_2025_1467_MOESM1_ESM.docx]

**Supplementary Information**

**Comparative phytochemical evaluation and authentication of four *Artemisia* species using integrated GC-MS, HPTLC and NIR spectroscopy approach**

**Ingy I. Abdallah^1,*^, Hebaalla A. Mahmoud^1^, Nadia A. El-Sebakhy^1^, Yasmin A. Mahgoub^1^**

**^1^ Department of Pharmacognosy, Faculty of Pharmacy, Alexandria University, Egypt.**

**^*^ Corresponding author at: Alkhartoom square, Department of Pharmacognosy, Faculty of Pharmacy, Alexandria University, Egypt, Alexandria 21521, Egypt.**

**E-mail:** [**ingy.ibrahim@alexu.edu.eg**](mailto:ingy.ibrahim@alexu.edu.eg) **(Ingy I. Abdallah)**


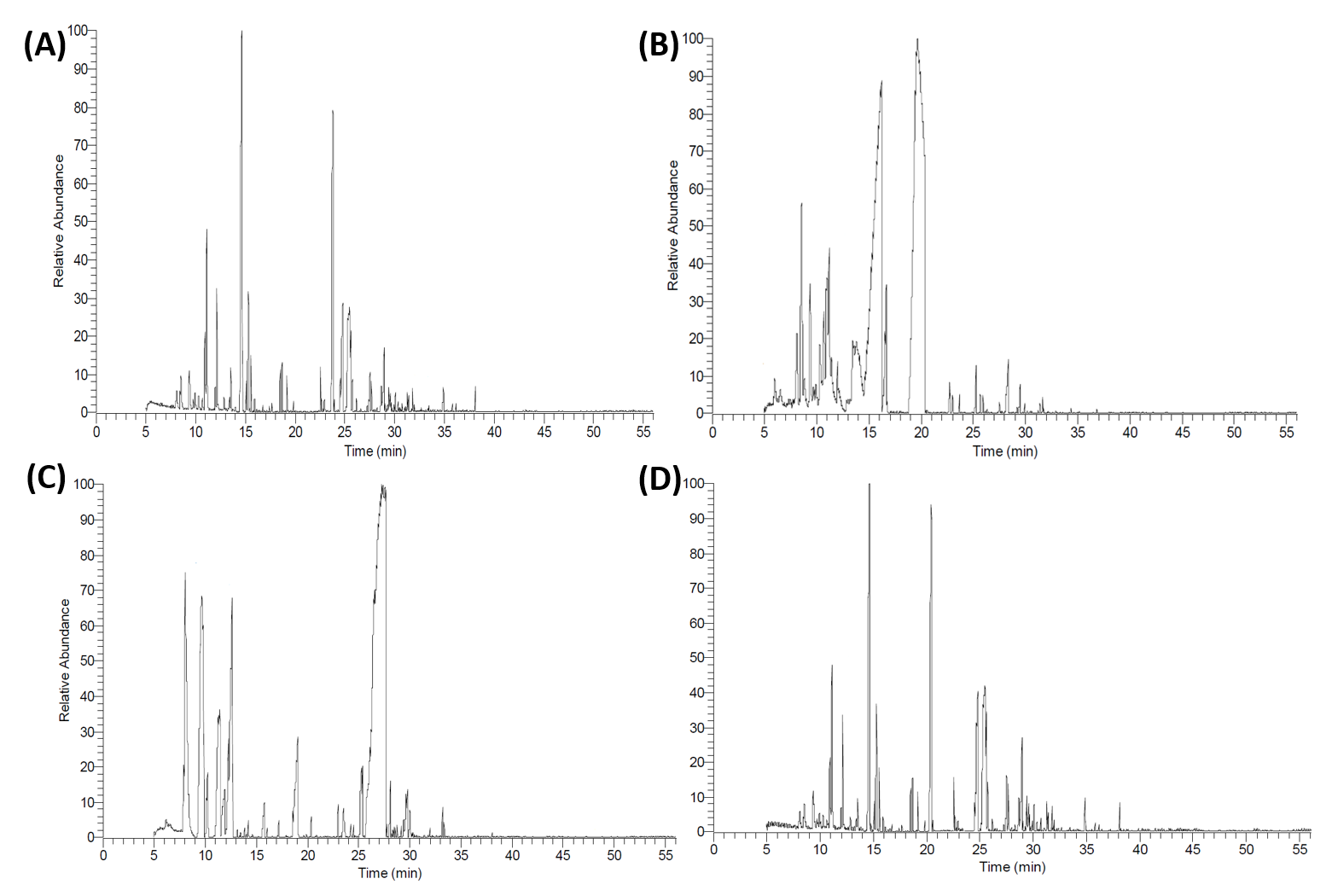


**Fig. S1. GC chromatogram of the volatile oil of (A) *A. annua*, (B) *A. herba-alba*, (C) *A. monosperma*, (D) *A. judaica***


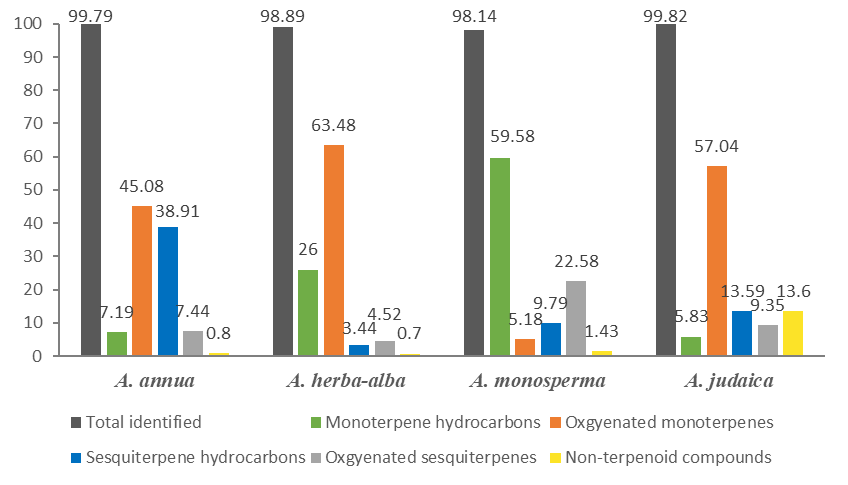


**Fig. S2. Percentages of the main classes of compounds identified in the volatile oils of the studied *Artemisia* species**


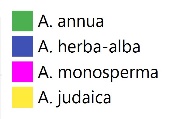

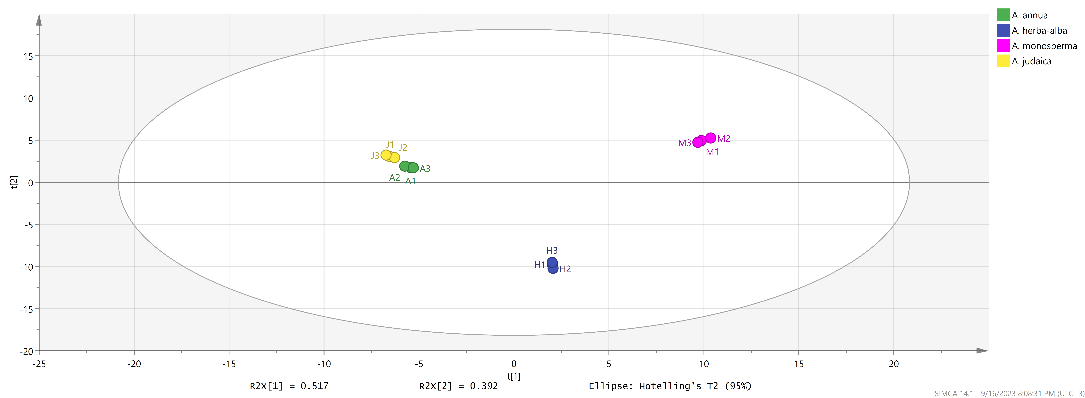


**Fig. S3. Score scatter plot of PCA model of volatile oils of the studied *Artemisia* species**

**
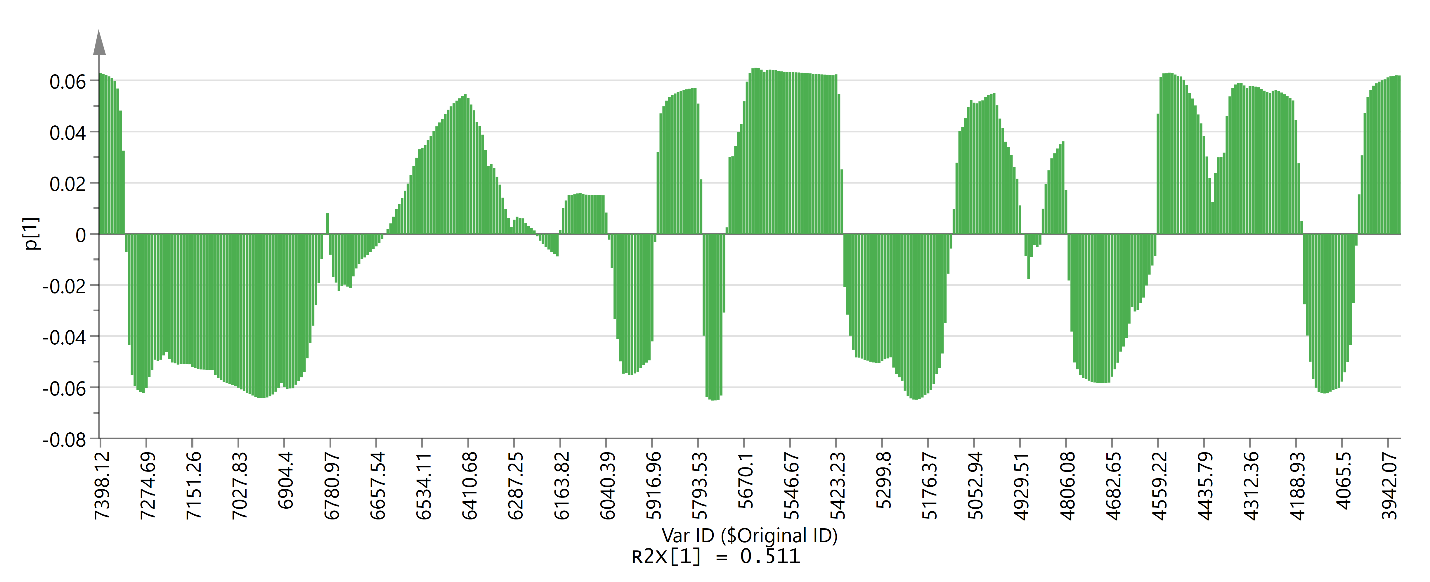
**

**Fig. S4. Loading plot corresponding to the PCA score scatter plot of the studied *Artemisia* species powders based on their NIR spectra**

| **A** | 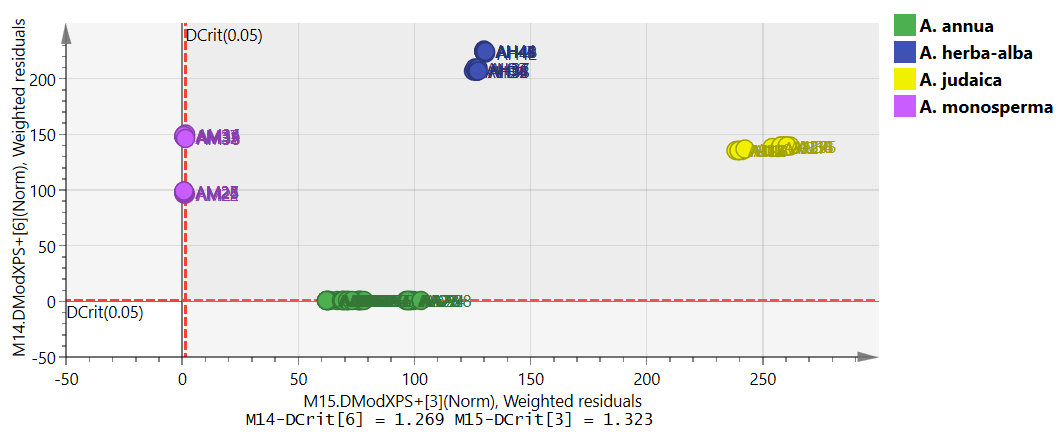 | 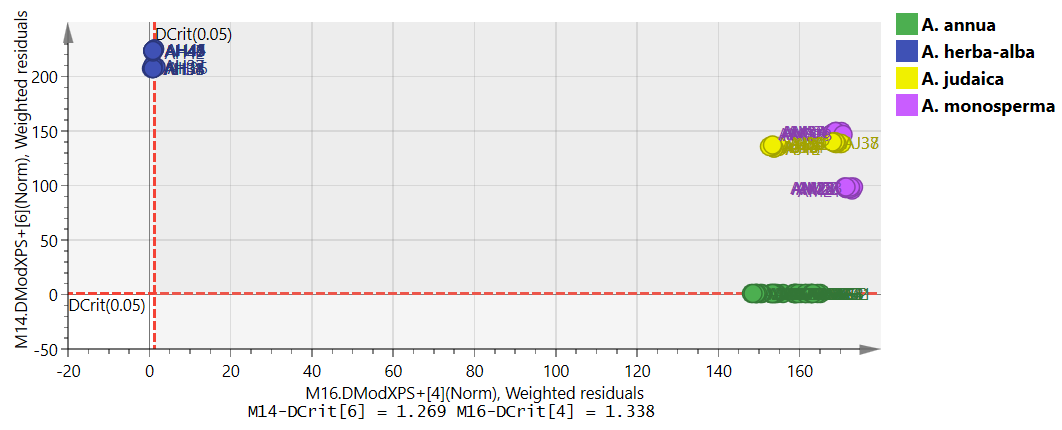 | 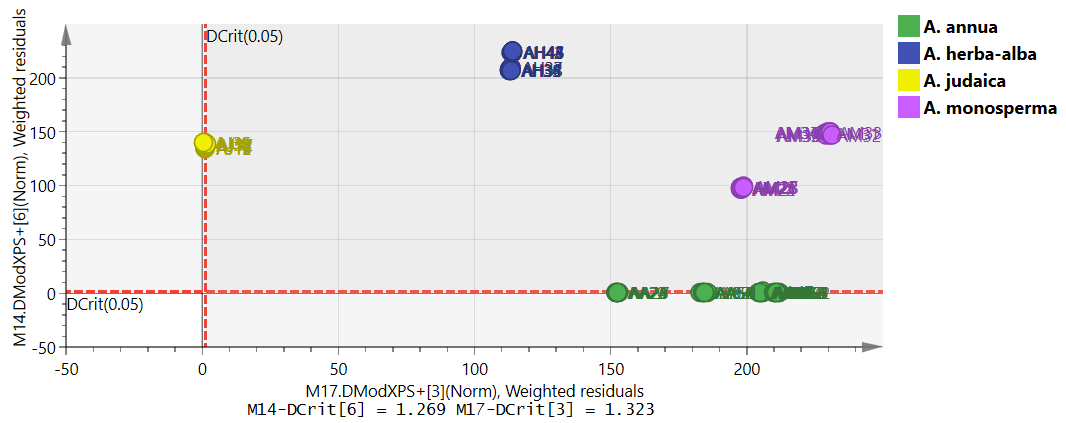 |
| --- | --- | --- | --- |
| **B** | 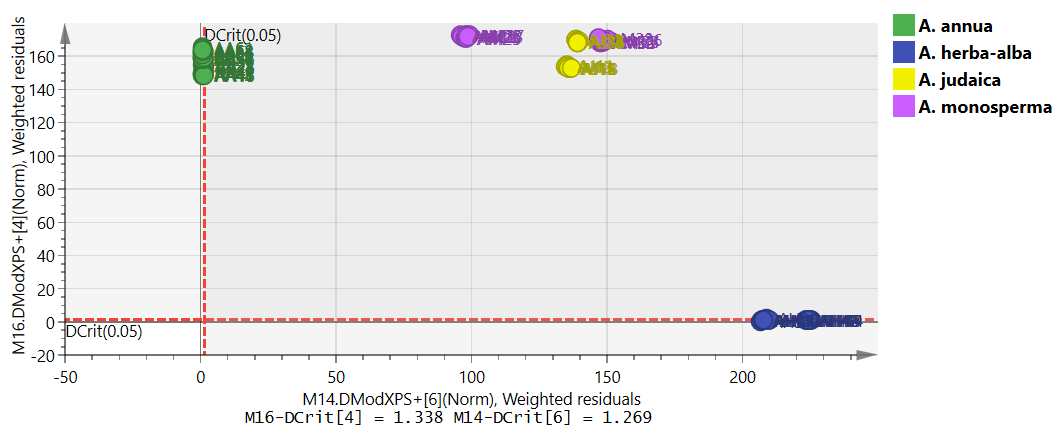 | 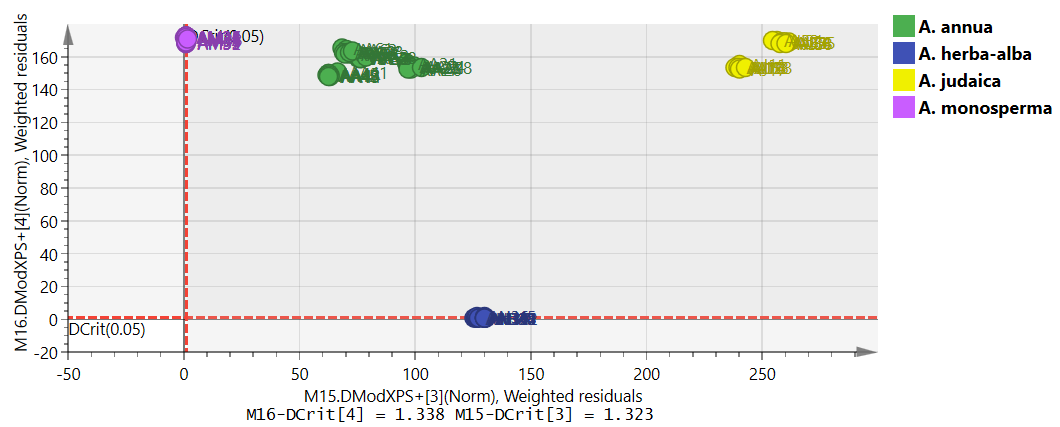 | 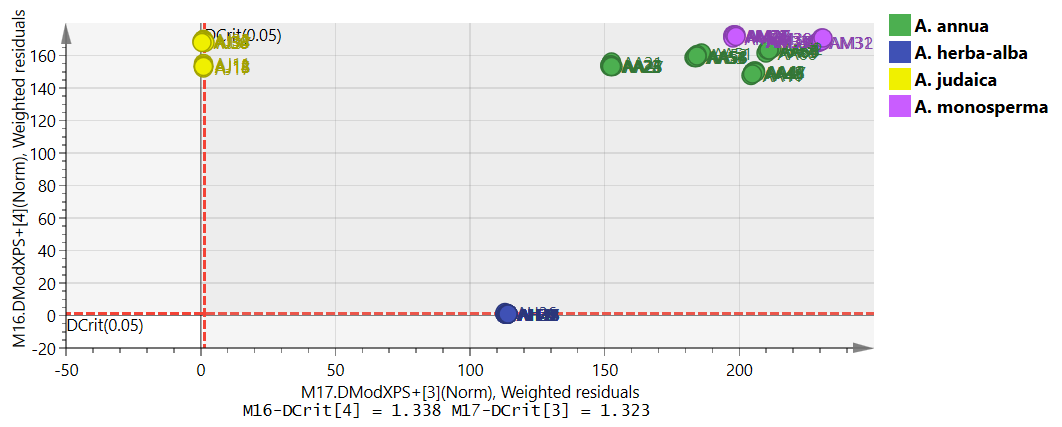 |
| **C** | 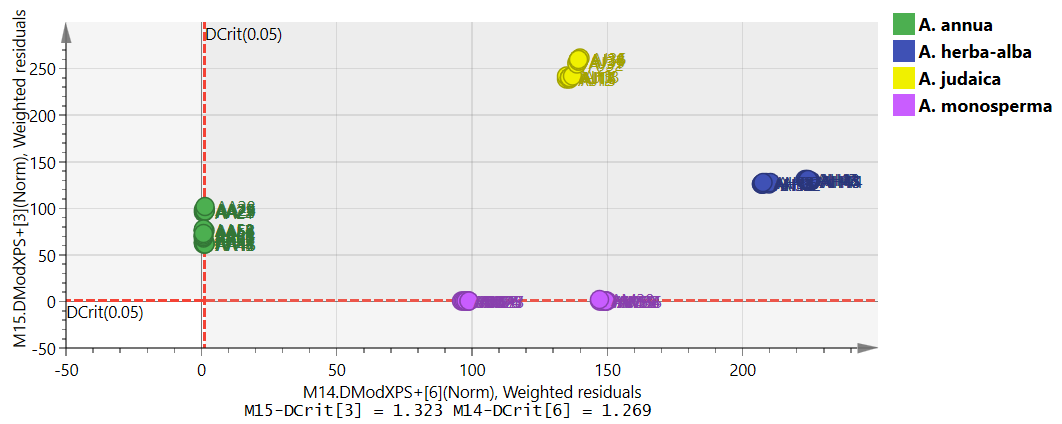 | 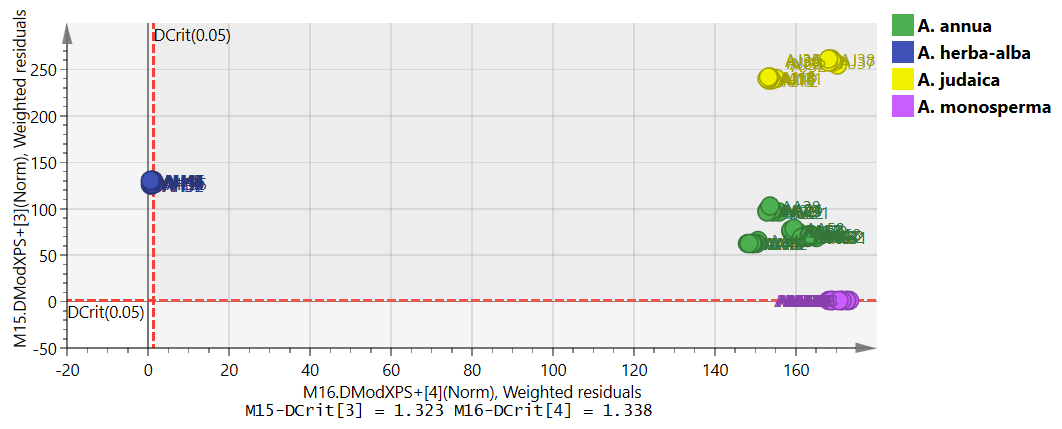 | 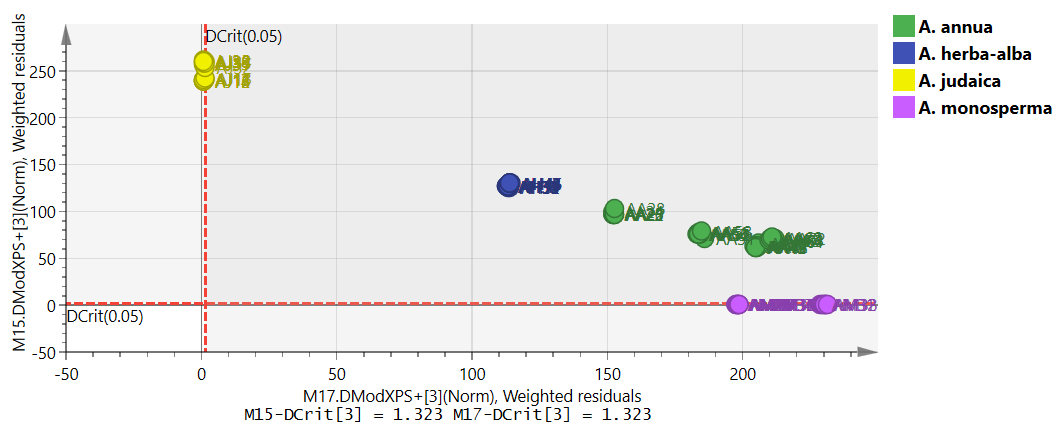 |
| **D** | 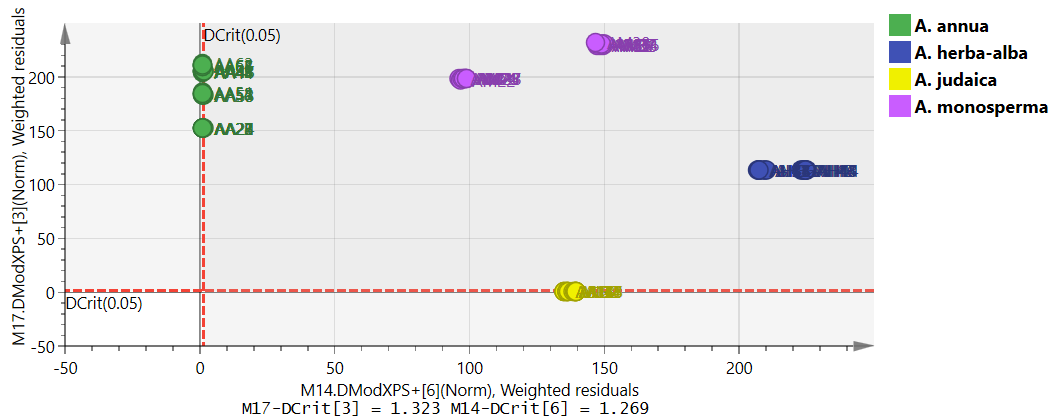 | 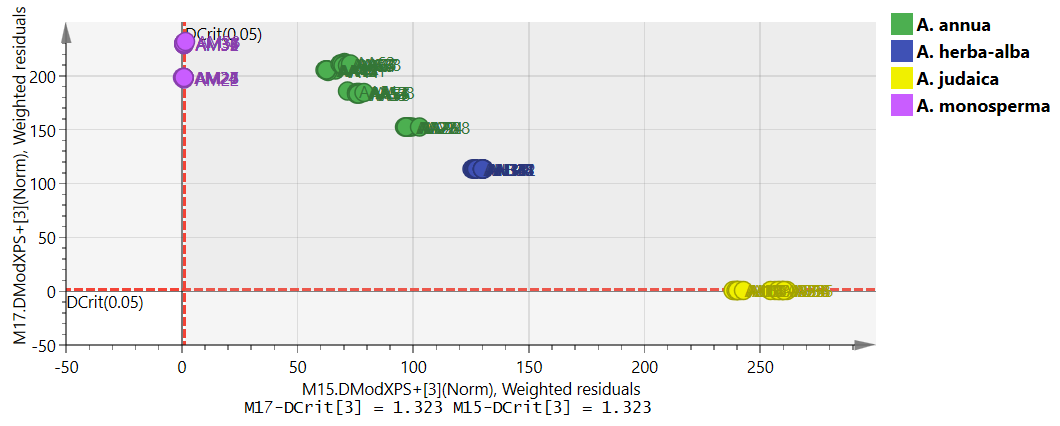 | 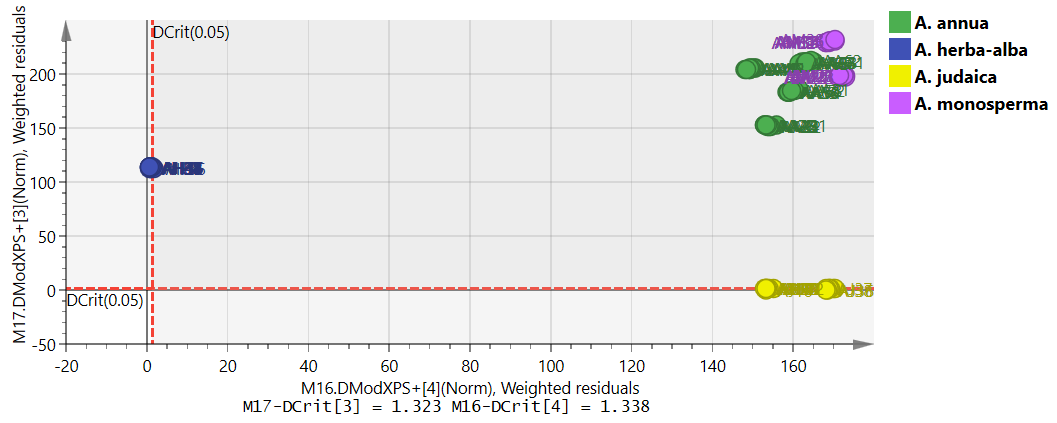 |
| **Fig. S5. Cooman’s plot for classification of each Artemisia species (x-axis) and each of the other species (y-axis) (A*) A. annua*, (B) *A. herba-alba*, (C*) A. monosperma*, (D) *A. judaica*** | | | |


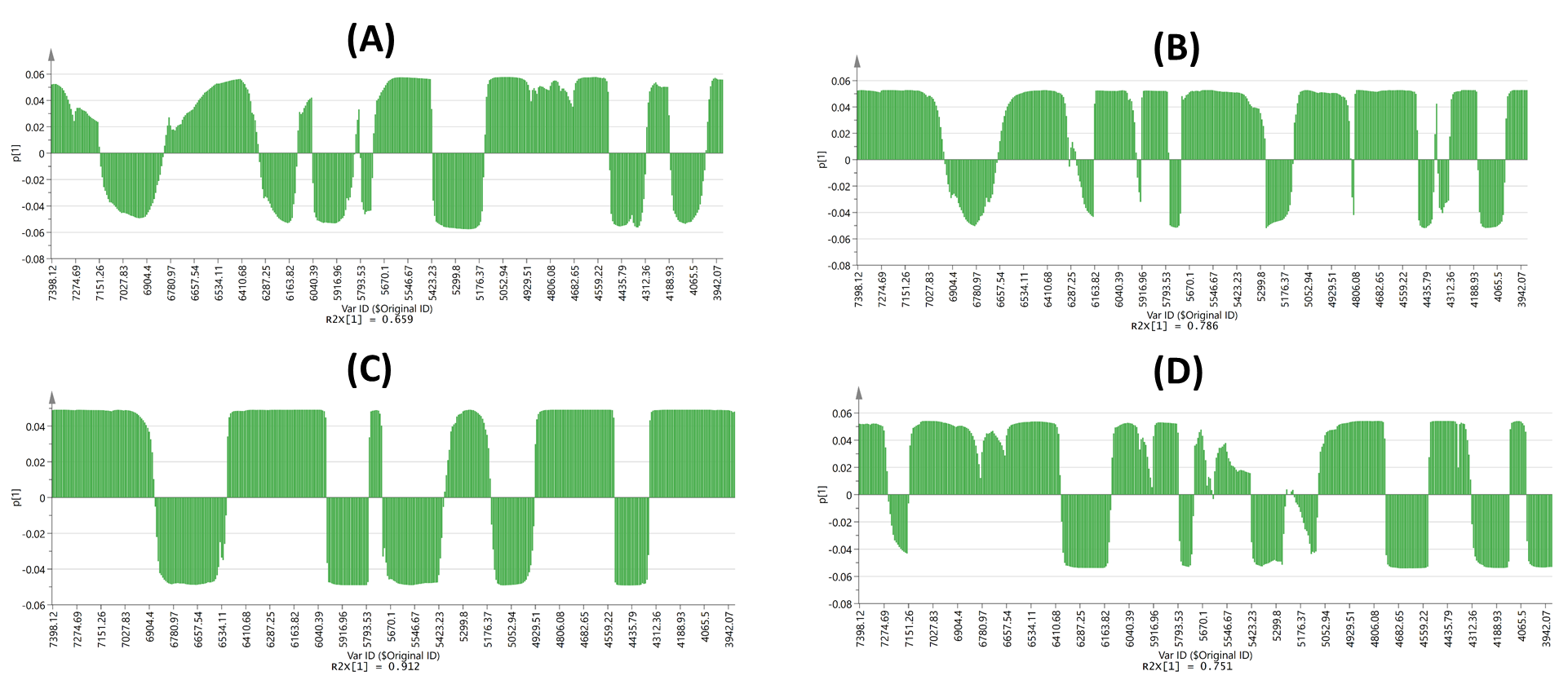
**Fig. S6. Loading plots corresponding to the SIMCA score scatter plot of (A) *A. annua* model, (B) *A. herba-alba* model, (C) *A. monosperma* model and (D) *A. judaica* model based on the NIR spectra of their powder samples.**

**Table S1. SIMCA class prediction for *Artemisia* species with full spectra (3800-7500 cm^-1^)**

|  | **Class** | **NS** | **Correct** | ***A. annua*** | ***A. herba-alba*** | ***A. monosperma*** | ***A. judaica*** | **No class** |
| --- | --- | --- | --- | --- | --- | --- | --- | --- |
| **Training (calibration) set** | ***A. annua*** | 30 | 100% | 30 | 0 | 0 | 0 | 0 |
|  | ***A. herba-alba*** | 15 | 100% | 0 | 15 | 0 | 0 | 0 |
|  | ***A. monosperma*** | 15 | 100% | 0 | 0 | 15 | 0 | 0 |
|  | ***A. judaica*** | 15 | 100% | 0 | 0 | 0 | 15 | 0 |
|  | **No class** | 0 | 0% | 0 | 0 | 0 | 0 | 0 |
| **Test set** | ***A. annua*** | 14 | 100% | 14 | 0 | 0 | 0 | 0 |
|  | ***A. herba-alba*** | 7 | 100% | 0 | 7 | 0 | 0 | 0 |
|  | ***A. monosperma*** | 7 | 100% | 0 | 0 | 7 | 0 | 0 |
|  | ***A. judaica*** | 7 | 100% | 0 | 0 | 0 | 7 | 0 |
|  | **No class** | 0 | 0% | 0 | 0 | 0 | 0 | 0 |

**Table S2. Details of collected plant samples for NIR analysis**

| **Sample name** | **Sample code** | **Region of collection** | | **Time of collection** |
| --- | --- | --- | --- | --- |
| ***A. annua*** | AA11 | Experimental garden of Faculty of Pharmacy, Cairo University, Giza, Egypt | Zone 1 | May 2021 |
|  | AA12 |  | Zone 1 | May 2021 |
|  | AA13 |  | Zone 1 | May 2021 |
|  | AA14 |  | Zone 1 | May 2021 |
|  | AA15 |  | Zone 1 | May 2021 |
|  | AA16 |  | Zone 1 | May 2021 |
|  | AA17 |  | Zone 1 | May 2021 |
|  | AA18 |  | Zone 1 | May 2021 |
|  | AA21 |  | Zone 2 | May 2021 |
|  | AA22 |  | Zone 2 | May 2021 |
|  | AA23 |  | Zone 2 | May 2021 |
|  | AA24 |  | Zone 2 | May 2021 |
|  | AA25 |  | Zone 2 | May 2021 |
|  | AA26 |  | Zone 2 | May 2021 |
|  | AA27 |  | Zone 2 | May 2021 |
|  | AA28 |  | Zone 2 | May 2021 |
|  | AA31 |  | Zone 3 | May 2021 |
|  | AA32 |  | Zone 3 | May 2021 |
|  | AA33 |  | Zone 3 | May 2021 |
|  | AA34 |  | Zone 3 | May 2021 |
|  | AA41 |  | Zone 4 | May 2021 |
|  | AA42 |  | Zone 4 | May 2021 |
|  | AA43 |  | Zone 4 | May 2021 |
|  | AA44 |  | Zone 4 | May 2021 |
|  | AA45 |  | Zone 4 | May 2021 |
|  | AA46 |  | Zone 4 | May 2021 |
|  | AA47 |  | Zone 4 | May 2021 |
|  | AA48 |  | Zone 4 | May 2021 |
|  | AA51 |  | Zone 1 | May 2022 |
|  | AA52 |  | Zone 1 | May 2022 |
|  | AA53 |  | Zone 1 | May 2022 |
|  | AA54 |  | Zone 1 | May 2022 |
|  | AA55 |  | Zone 1 | May 2022 |
|  | AA56 |  | Zone 1 | May 2022 |
|  | AA57 |  | Zone 1 | May 2022 |
|  | AA58 |  | Zone 1 | May 2022 |
|  | AA61 |  | Zone 2 | May 2022 |
|  | AA62 |  | Zone 2 | May 2022 |
|  | AA63 |  | Zone 2 | May 2022 |
|  | AA64 |  | Zone 2 | May 2022 |
|  | AA65 |  | Zone 2 | May 2022 |
|  | AA66 |  | Zone 2 | May 2022 |
|  | AA67 |  | Zone 2 | May 2022 |
|  | AA68 |  | Zone 2 | May 2022 |
| **Sample name** | **Sample code** | **Region of collection** | | **Time of collection** |
| ***A. herba-alba*** | AH11 | Alexandria northern coast kilo 90, Egypt | Zone 1 | May 2022 |
|  | AH12 |  | Zone 1 | May 2022 |
|  | AH13 |  | Zone 1 | May 2022 |
|  | AH14 |  | Zone 1 | May 2022 |
|  | AH31 |  | Zone 3 | May 2021 |
|  | AH32 |  | Zone 3 | May 2021 |
|  | AH33 |  | Zone 3 | May 2021 |
|  | AH34 |  | Zone 3 | May 2021 |
|  | AH35 |  | Zone 3 | May 2021 |
|  | AH36 |  | Zone 3 | May 2021 |
|  | AH37 |  | Zone 3 | May 2021 |
|  | AH38 |  | Zone 3 | May 2021 |
|  | AH41 |  | Zone 4 | May 2021 |
|  | AH42 |  | Zone 4 | May 2021 |
|  | AH43 |  | Zone 4 | May 2021 |
|  | AH44 |  | Zone 4 | May 2021 |
|  | AH45 |  | Zone 4 | May 2021 |
|  | AH46 |  | Zone 4 | May 2021 |
|  | AH47 |  | Zone 4 | May 2021 |
|  | AH48 |  | Zone 4 | May 2021 |
| ***A. monosperma*** | AM21 | Alexandria northern coast kilo 83, Egypt | Zone 2 | May 2021 |
|  | AM22 |  | Zone 2 | May 2021 |
|  | AM23 |  | Zone 2 | May 2021 |
|  | AM24 |  | Zone 2 | May 2021 |
|  | AM25 |  | Zone 2 | May 2021 |
|  | AM26 |  | Zone 2 | May 2021 |
|  | AM27 |  | Zone 2 | May 2021 |
|  | AM28 |  | Zone 2 | May 2021 |
|  | AM31 |  | Zone 3 | May 2021 |
|  | AM32 |  | Zone 3 | May 2021 |
|  | AM33 |  | Zone 3 | May 2021 |
|  | AM34 |  | Zone 3 | May 2021 |
|  | AM35 |  | Zone 3 | May 2021 |
|  | AM36 |  | Zone 3 | May 2021 |
|  | AM37 |  | Zone 3 | May 2021 |
|  | AM38 |  | Zone 3 | May 2021 |
|  | AM41 |  | Zone 4 | May 2022 |
|  | AM42 |  | Zone 4 | May 2022 |
|  | AM43 |  | Zone 4 | May 2022 |
|  | AM44 |  | Zone 4 | May 2022 |
|  |  |  | |  |
| **Sample name** | **Sample code** | **Region of collection** | | **Time of collection** |
| ***A. judaica*** | AJ11 | Red Sea Governorate, Egypt | Zone 1 | May 2021 |
|  | AJ12 |  | Zone 1 | May 2021 |
|  | AJ13 |  | Zone 1 | May 2021 |
|  | AJ14 |  | Zone 1 | May 2021 |
|  | AJ15 |  | Zone 1 | May 2021 |
|  | AJ16 |  | Zone 1 | May 2021 |
|  | AJ17 |  | Zone 1 | May 2021 |
|  | AJ18 |  | Zone 1 | May 2021 |
|  | AJ31 |  | Zone 3 | May 2021 |
|  | AJ32 |  | Zone 3 | May 2021 |
|  | AJ33 |  | Zone 3 | May 2021 |
|  | AJ34 |  | Zone 3 | May 2021 |
|  | AJ35 |  | Zone 3 | May 2021 |
|  | AJ36 |  | Zone 3 | May 2021 |
|  | AJ37 |  | Zone 3 | May 2021 |
|  | AJ38 |  | Zone 3 | May 2021 |
|  | AJ41 |  | Zone 4 | May 2022 |
|  | AJ42 |  | Zone 4 | May 2022 |
|  | AJ43 |  | Zone 4 | May 2022 |
|  | AJ44 |  | Zone 4 | May 2022 |
